# Promoting collective precycling behavior: results from a group intervention with Berlin households in Germany

**DOI:** 10.3389/fpsyg.2024.1340305

**Published:** 2024-06-11

**Authors:** Klara Wenzel

**Affiliations:** Center Technology and Society, Technische Universität Berlin, Berlin, Germany

**Keywords:** precycling, packaging waste prevention, pro-environmental behavior, social identity, collective action, field experiment, living lab

## Abstract

To tackle the global waste crisis, there is an urgent need for decisive and joint action at multiple levels. The collective behavior of a community could make a significant contribution. This paper presents the results of a field experiment designed to promote packaging waste prevention – called precycling – in a newly formed community setting, in Berlin, Germany. The aim was to examine the effect of the intervention on precycling and to examine the underlying social identity processes. Over a four-week period, 132 participants from 96 different households digitally received a combination of different interventions, that were theoretically informed by the Social Identity Model of Pro-Environmental Action (SIMPEA). Households were assigned to two intervention groups and a waiting control group. Data was collected before, immediately after and four months after the intervention to assess the impact of the intervention using multilevel models. After the intervention, the overall precycling behavior increased significantly, but not as a result of the different group conditions. In the more comprehensive intervention group, which included social interaction and behavioral experimentation, the community identification was strengthened and the reuse behavior, as a subset of precycling, increased. While a number of social identity processes (collective efficacy beliefs, having a precycling action goal, crisis appraisal, and sufficiency attitudes) were found to positively predict the precycling behavior, surprisingly, the predictive power of social norms and ingroup identification could not be confirmed. Overall, the presented community intervention promoted precycling. However, in this dynamic real-world setting, not all intervention elements worked as expected. The pitfalls and opportunities of this intervention are discussed, and ideas for translating the results into everyday precycling activities are presented.

## 1 Introduction

Increasing amounts of packing waste is associated with excessive resource consumption, climate-damaging emissions and environmental pollution (e.g., Wilson et al., 2015; Galloway and Lewis, 2016; Royer et al., 2018). This dramatic situation states a global phenomenon resulting from collective human behavior and is largely consensually perceived as a serious problem, especially the case of plastic packaging and waste (e.g., Heidbreder et al., 2019; The United Nations Environment Assembly, 2022). There is a serious need for decisive and joint action at different levels to tackle this crisis. Normalizing precycling behavior in households could contribute to this. Precycling describes behaviors aiming at packaging waste prevention such as rejecting waste caused by single use packaging, reducing packaging waste and reusing packaging (Klug and Niemand, 2021). However, the mere appraisal of this crisis does not lead to strong pro-environmental action against packaging waste due to, convenience, lack of knowledge and opportunities, strong habits, and also social factors, among others (Heidbreder et al., 2019; Wiefek et al., 2021). To date, many psychological studies on (packaging) waste prevention focus on individual factors of behavior change, e.g., individual knowledge, personal motives and norms, costs and benefits or morality (Ertz et al., 2017; Heidbreder et al., 2019, 2022; Kaplan Mintz et al., 2019; Heidbreder and Schmitt, 2020). These approaches disregard, that packaging waste prevention (like many other behaviors) is strongly interconnected with social processes: in multi-person households, people share routines and interact in terms of nutrition, food supply and thus also in respect of packaging waste. Social groups outside the household, for example pro-environmental communities, can also influence the success of precycling, e.g., through in-group norms, but also through group activities and social support in developing avoidance strategies (Wenzel and Süßbauer, 2021). Therefore, the dynamics of social identity processes should be taken into account in order to implement successful behavioral interventions for packaging waste prevention. The Social Identity Model of Pro-Environmental Action (SIMPEA, Fritsche et al., 2018) is strongly rooted in theory and can provide guidance in this regard. In the context of SIMPEA, precycling can be interpreted as a pro-environmental individual and collective behavior in response to the packaging waste crisis, embedded in social dynamics and shaped by social identity factors. Although recommendations have been made for possible intervention strategies in line with the social identity approach (Fielding and Hornsey, 2016; Fritsche et al., 2018) little is known about how they affect behavior change in real-life settings. To learn more about their effectiveness and conditions for success, field research is needed (Fielding and Hornsey, 2016). Living labs could be one promising methodological approach in this direction. Furthermore, interdisciplinary collaboration is proposed to help develop behavior-based solutions that reflect the complexity of the packaging waste problem (Heidbreder et al., 2019).

This paper is a response to some of these shortcomings. It presents results from an intervention called “Precycling-HomeLabs,” which aims to promote domestic precycling behavior in Berlin households (Germany), in the long and short term. The intervention concept understands precycling as individual and collective behavior and as an object of social dynamics, based on the theoretical principles of the SIMPEA. The first is to assess the impact of the different intervention strategies on precycling behavior. Then, it will be tested whether the psychological processes of ingroup identification, precycling-friendly ingroup norms, and collective efficacy beliefs predict precycling behavior. Another objective is to see if the participants would develop a sense of pro-environmental group identity through this top down community.

In the following, I describe the SIMPEA framework, the HomeLab concept and the hypotheses I derived from theory (Sections 2.1 to 2.3), the research design as well as the analytic strategy (Section 3). Furthermore, I report (section 4) and discuss the results and then draw implications for promoting precycling and the pro-environmental use of resources (Section 5).

## 2 Conceptual background and hypothesis development

### 2.1 The social identity model of pro-environmental action in the context of precycling

Fritsche et al. (2018) developed the social identity model of pro-environmental action (SIMPEA) based on an extensive review of the social identity perspective. Social identity “makes group behavior possible” (Turner, 1982, p. 21), especially when a group membership is present. Individuals who have internalized a respective group membership as part of their self can think and act as members of this ingroup (Tajfel and Turner, 1979). The SIMPEA emphasizes this potential of collective thinking and acting to promote pro-environmental behavior and provides a systematic approach to investigate social identity as driver of people’s appraisal and response to collective environmental crisis. Namely ingroup identification, ingroup norms and goals, and collective efficacy beliefs are proposed to decisively influence environmental action, together with emotions and motivations.

#### 2.1.1 Environmental crisis and response

The existence of an environmental crisis, together with the appraisal of this crisis, is the starting point of the SIMPEA. The immense quantities of packaging waste display a frighteningly visible environmental crisis that is widely appraised as such (Heidbreder et al., 2019; Menzel et al., 2021). Evidently, there is a need to respond to this packaging waste crisis. SIMPEA defines a response as an individual or collective pro-environmental behavior. Collective behavior represents private or public group-based behavior that is realized by a salient ingroup (Fritsche et al., 2018). In this study, doing precycling is the pro-environmental behavior to counteract the crisis. The term precycling refers to behavioral strategies that help prevent packaging waste by rejecting, reducing or reusing packaging, resulting in less resource use (Klug and Niemand, 2021; Wenzel and Süßbauer, 2021). One key question of this study is: how can precycling action be promoted and which role play social identity processes? As suggested by the model and some empirical research on waste prevention the factors ingroup identification, ingroup norms and goals, collective efficacy beliefs and motivations may determine precycling.

#### 2.1.2 Ingroup identity

People can experience ingroup identification at different levels, “any self-relevant group” (Fritsche et al., 2018, p. 259) can become a reference point for group-related self-definition. Social interaction in small groups (e.g., group discussions) seems to be one way to stimulate such a collective identity and might promote collective action (Postmes et al., 2005; Kuppens et al., 2013; Thomas et al., 2016a). By discussing a group-relevant topic, such as everyday precycling, one could create places of social connection and identification (Fritsche and Masson, 2021; Vesely et al., 2021), which in turn may promote pro-environmental action. Further, socially visible precycling behavior can connect individuals as members of a community of shared purpose (Cherrier, 2006,) who all support waste reduction. In the context of this study the term group is used to refer to “two or more individuals who are connected by and within social relationships” (Forsyth, 2010, p. 3), within the Precycling community, which was established with the goal of promoting pre-cycling. The identification with this pro-environmental community is expected to predict precycling behavior.

#### 2.1.3 Ingroup norms and goals

Groups are characterized by ingroup norms, which are shared rules and standards that reflect the members’ knowledge of what most other group members do (descriptive norms) and what they think one should (not) do (injunctive norms) (Cialdini et al., 1990). Ingroup norms also indicate the group’s goals (Fritsche et al., 2018). Providing information on social norms is a popular and widely used approach to encourage resource conservation (Abrahamse and Steg, 2013). Social norms have been shown to positively influence waste minimization behavior (de Groot et al., 2013; van der Linden, 2015; Heidbreder et al., 2019, 2022; Kaplan Mintz et al., 2019). To encourage pro-environmental actions it is recommended to emphasize existing pro-environmental group norms and to set normative group-goals (Fielding and Hornsey, 2016; Fritsche et al., 2018), e.g., by framing precycling as a group task. It was also recommended that normative interventions should be combined with information to achieve a greater impact on waste prevention (Heidbreder et al., 2019). Given this, I expect that social norms will also positively influence precycling behavior.

#### 2.1.4 Collective efficacy beliefs

Collective efficacy beliefs have been described as peoples’ beliefs in the collective capacity of a group to reach group goals and they are suggested to foster the group’s commitment to pursue shared goals (Bandura, 2000). Furthermore, they are proposed as critical for overcoming individual inaction and initiating collective action (Fritsche et al., 2018). In the context of waste, collective efficacy has been shown to predict environmental action (e.g., intended reductions in household waste, Morton et al., 2011, Study 2) and a study on plastic prevention points to the relevance of collective efficacy beliefs for achieving plastic reduction goals of moderate difficulty (Reese and Junge, 2017). Given these results, it is reasonable to assume that collective efficacy beliefs also influence precycling behavior.

#### 2.1.5 Sufficiency attitude as a motivating factor

Motivations are also seen to play a crucial role in collective pro-environmental action (Fritsche et al., 2018). They can lead to goal-directed behavior by energizing action and providing direction (Kleinginna and Kleinginna, 1981; Lu and Schuett, 2014). When persons appraise a crisis to be relevant to themselves or their ingroup, this can initiate personal and/or collective motivations. Such motivation can be a catalyst for collective processes (Fritsche et al., 2018). A sufficiency attitude may be a relevant source of motivation that influences the response to the packaging waste crisis. In the domain of food consumption sufficiency attitude was identified as a motivating factor with the strongest explanatory power (Verfuerth et al., 2019). Furthermore, Klug and Niemand (2021, p. 7) identified the consumer motivations “voluntary simplicity” and “low materialism” to predict precycling. These concepts show substantive intersections with the principles of sufficiency. Sufficiency in terms of “enoughness” (Tröger and Reese, 2021, p. 828) is a sustainability strategy that could contribute to reducing resource consumption through voluntary changes in environmentally relevant behavior patterns (Stengel, 2011). Further, sufficiency orientation was revealed as a significant predictor of plastic-free shopping (Heidbreder et al., 2022), and according to Wiefek et al. (2021), the implementation of sufficiency lifestyle represents “one way to reduce the consumption of plastic packaging.” Based on their observations that packaging in the food sector is mainly associated with animal products (e.g., dairy) and non-regional, non-seasonal products, they propose that sufficiency in terms of renunciation of packed products would reduce packaging waste (Wiefek et al., 2021). These results are similar to the findings by Wenzel and Süßbauer (2021), who identified a form of precycling characterized by renunciation, which means that people choose to abstain from certain packed products. Based on these findings and in response to the question by Reese et al. (2020) of how to better integrate sufficiency orientation into environmental psychological theorizing, I propose to consider sufficiency orientation as motivating factor for precycling behavior.

### 2.2 Behavioral experimentation in real-world settings

Finding robust and sustainable responses to pressing and complex environmental and societal challenges – such as the global packaging waste crisis – requires new formats, that transcend disciplinary boundaries and bring together researchers, civil society organizations, and private individuals (Beaudoin et al., 2022; Kofler, 2023). So-called real-world laboratories (e.g., Heiskanen et al., 2018) and living labs (e.g., Liedtke et al., 2012; Salter and White, 2013) are two formats that have become increasingly important to stimulate socio-ecological transformations (von Geibler, 2013; Kofler, 2023). Approaches such as these have been implemented to promote resource conservation behavior (Davies and Doyle, 2015; Devaney and Davies, 2016; Breadsell J. et al., 2019b; Breadsell J. K. et al., 2019a). Davies and Doyle (2015) introduced the term “HomeLab” to describe a living lab intervention in a real home environment that aims to encourage sustainable practices through a package of interventions, such as experimentation with new behaviors, informational support, and self-observation (Devaney and Davies, 2016). The HomeLab concept was adapted to packaging prevention for this paper.

### 2.3 Objective of this research and hypotheses

This paper presents results from the intervention called “Precycling-HomeLabs.” The “Precycling-HomeLabs” were designed and conducted as an inter- and transdisciplinary field experiment in households and they are part of the project “PuR – Precycling as a means of resource efficiency – systemic solutions for packaging prevention.” The overall purpose was to investigate the effects of the HomeLabs on the generation of packaging waste in real-world households and to explore the conditions for changing behavior by combining methods from different scientific disciplines, namely sociology, psychology and environmental engineering.

In this article, I only report results from a psychological perspective. A description of the overall concept and preliminary findings from all disciplines were published in Süßbauer et al. (2023). Results from a life cycle assessment (LCA) of this approach can be found in Caspers et al. (2023).

The design of the intervention was to a large extent guided by the theoretical assumptions and recommendations of SIMPEA and by evidence-based social identity strategies for the promotion of pro-environmental behavior (e.g., Fielding and Hornsey, 2016; Fritsche et al., 2018, as described in Section 2.1). Further, evidence from previous studies on waste-prevention and resource conservation behavior was included (also described in Section 2.1). Additionally, the HomeLab concept (Davies and Doyle, 2015; Devaney and Davies, 2016) inspired the intervention (e.g., the Precycling Starter Kit, Section 3.1.2). The specific translation of these elements into the intervention is explained in the method Section 3.1.

The objective was, first, to quantitatively assess the specific short and long-term impact of the intervention on the participants’ precycling behavior in the different conditions to see which type of intervention strategies promote precycling behavior. A second objective was to see if the top-down created online community-setting would build an precycling-positive ingroup identity. Furthermore, it was of interest to test which social identity processes influence the intervention outcomes in this real-world context, namely, whether the proposed SIMPEA components ingroup identification, precycling-friendly ingroup norms and collective efficacy beliefs explain precycling behavior and ingroup identification. For this purpose, theory- and evidence-based hypotheses were formulated and tested. The hypotheses, sample size, exclusion criteria and planned analyses were preregistered.^1^

#### 2.3.1 Precycling behavior

*Hypothesis 1.1:* As a result of the interventions in the intervention groups, more precycling behavior (response) is realized in the intervention groups compared to the control group.

*Hypothesis 1.2:* Ingroup identification with the participants of the Precycling-HomeLabs positively predicts precycling behavior (response).

*Hypothesis 1.3:* Pro-precycling ingroup norms positively predict precycling behavior (response).

*Hypothesis 1.4:* Collective efficacy beliefs towards the participants of the Precycling-HomeLabs positively predict precycling behavior (response).

#### 2.3.2 Ingroup identification

*Hypothesis 2.1:* As a result of the interventions, the ingroup identification with the participants of the Precycling-HomeLabs becomes salient and increases in the intervention groups compared to the control group.

#### 2.3.3 Exploratory analysis

For exploratory purposes, I test the influence of the following SIMPEA factors on precycling behavior and reuse behavior: sufficiency attitude, precycling goal, appraisal of the packaging waste crisis.

## 3 Methods and material

### 3.1 Participants and design

The participants were recruited in Berlin with flyers at outdoor events, neighborhood offices and online. The flyer provided information on the project, the study and a link and QR code for the registration. The following was offered for participation: consultancy services and events on the subject of precycling, a “Precycling Starter Kit” with information and materials on precycling, an evaluation of the study results and an allowance between 50€ and 100€ per household after having participated fully. The allowance depended on the respective condition and the extent of their participation. Participants had to be at least 18 years old, live in Berlin, Germany, be capable of speaking, reading and writing German, and have regular access to the internet. People who had participated in a previously conducted interview study on precycling were not allowed to participate. Throughout the study, information on the procedure was communicated via e-mail.

In total, 132 participants from 96 households took part in the study. Since there exist no uniform recommendations for power analyses in complex multilevel designs yet (Bell et al., 2014), I estimated the necessary sample size with different approximations, first with a repeated measure ANOVA with within-between interaction, an effect size (*f* = 0.15), an alpha of 0.05, and three measurements in three groups. Second, I did an approximation with an ANCOVA assuming an effect size (*f* = 0.25), an alpha of 0.5, three intervention groups and 11 covariates, using G*Power3.1 (Faul et al., 2009). According to the ANOVA, a total sample of 117 participants was required to achieve a power of 0.90 and to detect a small to medium effect and according to the ANCOVA, a total sample of 158 participants was required to achieve a power of 0.80 [as defined by Cohen (1988)]. Third, according to a rule of thumb for the use of multiple regression described by Tabachnick and Fidell (2014), a sample size of 115 would have been appropriate for a model with 11 covariates. Though these different approaches do not correspond to the multilevel models used in the present study, they can be understood as approximations. For more specific information on sample size and power in multilevel models, see section 3.3.

The Precycling-HomeLabs were conducted as a field experiment with a between-group design including three different conditions, two intervention groups and one waiting control group. The households were randomly assigned to one of the three groups, ensuring that the different household types (e.g., family, single) were equally represented in all conditions (randomization with stratification regarding household type). The first intervention group (IG1) consisted of 46 participants, the second intervention group (IG2) comprised 41 participants and 45 participants had been assigned to the waiting control group (WCG).

Data was collected between May and November 2021. The main intervention period was from May 10th to June 6th. In May, before the intervention, 126 participants completed the first online survey (pre, t0). After the intervention, 90 participants responded to the second survey (post, t1) and 85 participants took part in the follow-up survey (t2). The waiting control group completed four surveys in total. The additional survey after the delayed intervention was completed by 24 participants of the waiting control group (t1b). Table 1 displays detailed sociodemographic information separately for the different intervention groups and all different times of measurement. In the multi-level regression analysis, 126 participants from 92 households were included (see Section 3.3 for inclusion criteria).

**Table 1:** Sociodemographic characteristics of the participants separately for the different intervention groups at the different times of measure (*N* = 132).

	Intervention group 1 (IG1)	Intervention group 2 (IG2)	Waiting control group (WCG)
Gender	t0: male = 12, female = 32	t0: male = 10, female = 29	t0: male = 15, female = 28
	t1: male = 11, female = 20	t1: male = 6, female = 20	t1: male = 10, female = 23
	–	–	t1b: male = 6, female = 18
	t2: male = 9, female = 19	t2: male = 7, female = 22	t2: male = 9, female = 19
Age	*M*_0_ = 37.52 ( *SD*_0_ = 11.27)	*M*_0_ = 38.97 (*SD*_0_ = 14.96)	*M*_0_ = 37.42 (*SD*_0_ = 13.33)
	*M*_1_ = 37.48 (*SD*_1_ = 11.03)	*M*_1_ = 37.12 (*SD*_1_ = 12.76)	*M*_1_ = 38.73 (*SD*_1_ = 12.56)
	-	-	*M_1b_* = 38.88 (*SD*_3_ = 13.99)
	*M*_2_ = 39.5 (*SD*_3_ = 11.56)	*M*_2_ = 38.31 (*SD*_3_ = 14.31)	*M*_2_ = 38.18 (*SD*_3_ = 13.97)
Household composition	t0: 22.7% single	t0: 17.9% single	t0: 14.0% single
	t0: 25.0% couple	t0: 20.5% couple	t0: 32.6% couple
	t0: 38.6% family	t0: 41.0% family	t0: 32.6% family
	t0: 13.6% flat-sharing	t0: 20.5% flat-sharing	t0: 20.9% flat-sharing
	t1: 19.4% single	t1: 23.1% single	t1: 15.2% single
	t1: 25.8% couple	t1: 26.9% couple	t1: 33.3% couple
	t1: 41.9% family	t1: 26.9% family	t1: 33.3% family
	t1: 12.9% flat-sharing	t1: 23.1% flat-sharing	t1: 18.2% flat-sharing
	–	–	t1b: 16.7% single
	–	–	t1b: 45.8% couple
	–	–	t1b: 16.7% family
	–	–	t1b: 20.8% flat-sharing
	t2: 14.3% single	t2: 25.0% single	t2: 17.9% single
	t2: 28.6% couple	t2: 21.4% couple	t2: 28.6% couple
	t2: 46.4% family	t2: 35.7% family	t2: 28.6% family
	t2: 10.7% flat-sharing	t2: 17.9% flat-sharing	t2: 25.0% flat-sharing
Household size	*M*_0_ = 2.57 (*SD*_0_ = 1.37)	*M*_0_ = 2.51 (*SD*_0_ = 1.17)	*M*_0_ = 2.51 (*SD*_0_ = 1.26)
	*M*_1_ = 2.59 (*SD*_1_ = 1.40)	*M*_1_ = 2.19 (*SD*_1_ = 0.98)	*M*_1_ = 2.31 (*SD*_1_ = 0.93)
	–	–	*M*_12_ = 2.17 (*SD*_12_ = 0.82)
	*M*_2_ = 2.89 (*SD*_2_ = 1.45)	*M*_2_ = 2.31 (*SD*_2_ = 1.20)	*M*_2_ = 2.23 (*SD*_2_ = 0.91)
Education	t0: 27.3% high school	t0: 28.2% high school	t0: 41.9% high school
	t0: 72.7% university degree	t0: 71.8% university degree	t0: 58.1% university degree
	t1: 35.5% high school	t1: 19.2% high school	t1: 30.3% high school
	t1: 64.5% university degree	t1: 80.8% university degree	t1: 69.7% university degree
	–	–	t1b: 37.5% high school
	–	–	t1b: 62.5% university degree
	t2: 28.6% high school	t2: 17.9% high school	t2: 40.7% high school
	t2: 71.4% university degree	t2: 82.1% university degree	t2: 59.3% university degree
Paid hours worked	*M*_0_ = 24.01 (*SD*_0_ = 16.42)	*M*_0_ = 24.79 (*SD*_0_ = 16.54)	*M*_0_ = 24.42 (*SD*_0_ = 14.59)
	*M*_1_ = 24.79 (*SD*_1_ = 16.57)	*M*_1_ = 25.00 (*SD*_1_ = 16.41)	*M*_1_ = 25.34 (*SD*_1_ = 13.19)
	–	–	*M*_1b_ = 24.96 (*SD*_12_ = 12.39)
	*M*_2_ = 25.65 (*SD*_2_ = 15.62)	*M*_2_ = 25.22 (*SD*_2_ = 16.43)	*M*_2_ = 24.27 (*SD*_2_ = 14.59)

The label high school degree summarizes the German degrees “Realschule,” “Fachabitur” and “Abitur.” The times of measure are labeled as t0 = pre Intervention, t1 = post intervention, t1b = post intervention in the WCG, and t2 = follow-up.

**Figure 1:**
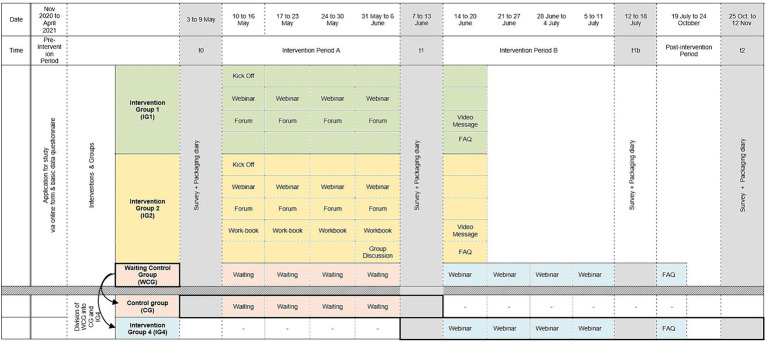
Study design with regard to temporal process, methods, different intervention groups and data restructuring of the waiting control group. t0 represents the pre measure before the intervention (n_0_=126), t1 represents the post measure (n_1_=90), t1b is the post measure in the waiting control group (n_1b_=24), and time t2 the follow-up measure (n_2_=85).

The study design is visualized with regard to the temporal process, methods and the intervention groups in Figure 1 and Table 2 gives a summary of formats applied in the different groups.

**Table 2:** Summary of intervention formats and measures in the different groups.

Intervention formats	Setting	Intended outcome	Implementation depending on group condition		
			IG1	IG2	WCG
Kick-off event	Plenary (online)	Creating an ingroup identity Making precycling-friendly ingroup norms salient Setting a collective action goal Emphasizing collective efficacy beliefs	✓	✓	
Webinars	Plenary (online) (recording in WCG)	Increasing knowledge about precycling behavior Increase precycling behavior	✓	✓	✓ (delayed)
Online forum	Individual (online)	Providing information Enabling social interaction	✓	✓	
Video message	Individual (online)	Refreshing action goal Giving information about study	✓	✓	
FAQ	Individual (online)	Increasing knowledge about precycling	✓	✓	✓ (delayed)
Precycling starter kit	Individual (analog)	Increasing knowledge about precycling Encouraging behavioral experiments		✓	
Group discussion	Small groups (online)	Creating an ingroup identity Enabling social interaction		✓	
Packaging diary	Individual (analog)	Encouraging behavioral self-observation and documentation of waste behavior	✓	✓	✓
**Measures**					
Online questionnaire	Individual (online)	Get information about participants	✓	✓	✓

#### 3.1.1 Basic intervention in group one and two

The participants in the intervention group (IG1) and intervention group (IG2) attended a four-week intervention between the 10th of May and the 6th of June. The period of the intervention started with a kick-off webinar and ended with a video message. Further, four webinars on precycling-related topics and access to an online forum were provided. The different aspects of the intervention will be described now:

##### 3.1.1.1 Kick-off-webinar

The kick-off webinar (45 min) was held via zoom by the PuR team researcher to officially launch the HomeLabs and introduce the team and the process. It represented the key element of the social identity intervention for IG1 and IG2. The team presentation addressed different elements of social identity that are thought to influence pro-environmental behavior and precycling (Fielding and Hornsey, 2016; Fritsche et al., 2018; Wenzel and Süßbauer, 2021), namely ingroup identity, collective efficacy beliefs, ingroup norms, and ingroup goals:

- **Creating an ingroup identity**. The presentation framed the precycling action during the HomeLabs as a collective project (as proposed by Fritsche et al., 2018) and emphasized the participants’ proactive participation in the project for the upcoming weeks. Phrases like “we,” “our,” “together” should highlight the fact, that all participants and the PuR team are members of the Precycling-HomeLabs and that they will work, experiment, learn and share their experiences together. This was intended to create an ingroup identity.
- **Making precycling-friendly norms salient**. The aim was to make visible the precycling friendly norms that were already in place within the group of participants. To learn about participants’ norms and share them with the group at the kick-off, the t0 survey asked participants in an open response format about their reasons for participating in the Precycling HomeLabs. Some representative and illustrative quotes from these responses were then presented during the kick-off webinar (e.g., thinking about consumption patterns is important, avoiding waste makes the world a better place). By quoting, the group members should speak for themselves, as ingroup sources are more influential (Fielding and Hornsey, 2016; Fritsche et al., 2018).
- **Setting a collective action goal**. During the kick-off, doing precycling was formulated as the important goal of the HomeLab project. To emphasize this group goal and to “give the member’s actions direction” (Fritsche et al., 2018, p. 249), participants were invited to make an effort to reduce food-packaging waste in their households during the coming weeks of the study.
- **Emphasizing collective efficacy beliefs**. In the presentation, the Precycling-HomeLabs were presented as a community project in which the participants support the research on precycling and thus contribute to find solutions to the packaging waste crisis. This was to emphasize the collective efficacy beliefs related to precycling, as the emphasis on joint solutions is (Fritsche et al., 2018) is expected to promote pro-environmental action.

##### 3.1.1.2 Webinars

The webinars served to provide knowledge for action and also enabled the exchange of experiences. The participants of IG1 and IG2 were invited to take part in one zoom-webinar each week for a total of four weeks. Each webinar lasted between 60 and 90 min. In these webinars, information on four different precycling topics was provided by experts. Through interactive elements the participants were invited to share their experiences (e.g., menti.com), to ask questions and to discuss the content. The interactive elements should also strengthen the ingroup identity. Participants who could not attain the webinar synchronically were provided the recording. Please find the briefly sketched content of the webinars in the Appendix A.

##### 3.1.1.3 Online forum

The participants in IG1 and IG2 had access to an online forum during the intervention period. In this online forum the recordings from the webinars were provided, and participants could exchange and interact with the other participants and ask questions to the community and to the project team.

##### 3.1.1.4 Video message

On the 14th of June, after the intervention period and the post-online survey (t1), the participants received a 14-min video message with a short retrospective summary of the collective activities of the last weeks. Again, in order for the participants to continue with their precycling behavior, the collective action goal of doing precycling was formulated. Finally, they received organizational information about the course of the study and a FAQ document with information on precycling.

##### 3.1.1.5 Packaging diary

The participants documented their generated household food-packaging waste with a paper-pencil packaging diary in a pre-structured template throughout one week at three, respectively, four (WCG) different times. The template can be found in Appendix B.

#### 3.1.2 Extended intervention in group two

In addition to the intervention procedure described in Section 3.1.1, participants in IG2 joined activities that provided space for social interaction within the community (group discussion) and aimed to stimulate specific precycling behaviors (reusing food containers). These socially vivid and behavior specific elements were included to see if this would booster the intervention effects, compared to the basic intervention:

##### 3.1.2.1 Group discussion

In the last week of the intervention period, the households in IG2 were invited to join a guided group discussion (Kitzinger, 1995; Kühn and Koschel, 2011) together with five other households lasting an average of about two hours (mean = 122 min). Among others, the participants talked about the workbooks, their packaging waste, precycling strategies, and precycling goals. The interactive discussions were designed to create a collective place of ingroup identification and to strengthen the sense of group membership through interacting and exchanging (Fritsche and Masson, 2021; Vesely et al., 2021). The discussion guide is presented in Appendix C.

##### 3.1.2.2 Precycling starter kit

The purpose of the kits was to provide information and to encourage precycling, informed by literature on practice-based interventions (Davies and Doyle, 2015; Devaney and Davies, 2016; Laakso, 2017). Each household in group two received one. They included flyer material with information on precycling, a reusable box for take-out food and a pre-structured workbook. The Workbook was informed by concepts by Langley et al. (2010), Scott et al. (2012), Hong et al. (2013), Kuijer (2014), Breadsell J. et al. (2019b), Breadsell J. K. et al. (2019a), and Breadsell and Morrison (2020). The purpose of the workbook was to encourage participants to do behavioral experiments in packaging waste prevention (e.g., shopping in zero waste shop, using reusable containers for take-away food) and to document the process. It also provided an opportunity for participants to reflect on their experiences during the HomeLabs, for example, by asking them to exchange experiences with household members and to reflect on what they learned from the webinars.

#### 3.1.3 Delayed and reduced intervention in waiting control group

Between t0 and t1, the participants in the WCG waited and received no intervention other than documenting their waste in the packaging waste diary. After t1, the WCG received a reduced intervention: they were provided with the recordings of the webinars. Therefore, they were send the link to one recording per week via e-mail. Afterwards, they received an e-mail with the information about the follow-up and the FAQ document with information on precycling.

### 3.2 Measures

During the study, several measures were assessed that are not related to the research questions of this article. Only the measures relevant to this paper are described below. The items of the questionnaires are provided in Appendix D.

#### 3.2.1 Online questionnaire

The participants completed three (intervention groups) or four (waiting control group) online questionnaires. The baseline questionnaire was provided to all participants one week before the intervention had started (t0 = 3rd to 9th of May), the second one was provided after the intervention period (t1 = 7th to 13th of June), and the third one was completed three months after the interventions (t2 = 25th to 12th of November). The participant in the waiting control group responded one more time to the online survey. As they were conceptualized as a waiting control group, they received the second questionnaire twice: once after the waiting period (t1), when the intervention groups have received their intervention, and once after the waiting control group has received its delayed intervention (t12). In this way, both the effect of the waiting time and the effect of the webinar intervention could be measured. We pretested the questionnaire for comprehensibility and adjusted the material according to the feedback.

The pre-intervention, post-intervention and follow-up questionnaires included the following measures in the order shown, with the exception of sufficiency attitude and appraisal, which were only measured in the pre-questionnaire. The response scales are based on the respective reference sources.

##### 3.2.1.1 Ingroup identification

Ingroup identification with the HomeLab participants was measured with the German Multidimensional and Multicomponent Measure of Social Identification by Roth and Mazziotta (2015). The measure consists of 15 items structured by the two dimensions *self-investment* (including centrality, solidarity, satisfaction) and *self-definition* (characterized by individual self-stereotyping, in-group homogeneity). Participants rated their agreement to the respective statements on a five-point Likert Scale (from 1 = “strongly disagree” to 5 = “strongly agree”). I computed scores on each sub-scale by averaging across items (*α*_t0_ = 0.91, *α*_t1_ = 0.94, *α*_t12_ = 0.93, *α*_t2_ = 0.94) for each participant. Higher scores indicate a stronger response towards identification.

##### 3.2.1.2 Precycling behavior

Precycling behavior was measured with the one-dimensional scale by Klug and Niemand (2021) consisting of five items and adapted for food packaging waste, for example “I consciously buy unpackaged food”. Each participant rated their agreement to the respective statements on a seven-point Likert scale (ranging from 1 = “completely disagree” to 7 = “completely agree”). An individual mean score for precycling behavior was calculated (*α*_t0_ = 0.85, *α*_t1_ = 0.85, *α*_t12_ = 0.81, *α*_t2_ = 0.89).

##### 3.2.1.3 Reuse behavior

As a specification of precycling behavior, I measured the reusing of packaging and reusable container with six items. I developed four items based on insights from Wenzel and Süßbauer (2021) and adapted two items from Kaplan Mintz et al. (2019) and Robertson and Barling (2013). I conducted a factor analysis to prove that the scale can be interpreted as unidimensional (see Section 4.1.). Participants were asked to rate the frequency of performing specific reusing behaviors (e.g., “I use reusable eating utensils when I am on the road”) on a five-point Likert scale (1 = “never” to 5 = “very often”). An individual mean score was calculated (*α*_t0_ = 0.55, *α*_t1_ = 0.52, *α*_t12_ = 0.58, *α*_t2_ = 0.56).

##### 3.2.1.4 Precycling goal

I equated the goal to implement precycling with the goal intention (Klöckner, 2014) to do precycling. Participants responded to the prospective statement “I plan to avoid food packaging waste” (Heidbreder et al., 2020) on a five-point Likert scale (from 1 = “strongly disagree” to 5 = “strongly agree”). To measure goal intention in the post and follow-up questionnaire, I, respectively, used the retrospective statement “for the past five weeks / past three month I have been trying to avoid food packaging waste”.

##### 3.2.1.5 Social norms

Descriptive social norms towards precycling of food packaging were assessed with one modified item by Reese and Junge (2017). Participants were invited to estimate the frequency with which the HomeLab participant try to minimize packaging waste when buying food using a 5-point Likert scale (from 1 = “never” to 5 = “very often”). Injunctive social norms were assessed with two modified items by Reese and Junge (2017). Participants were asked to rate the statements on a scale (1–“approve” to 7–“disapprove”, or 1–“irrelevant” to 7–“very relevant”), e.g.: “The HomeLab participants try to minimize packaging waste when shopping food” as (irrelevant – very relevant). The analysis is based on the individual mean score (*α*_t0_ = 0.74, *α*_t1_ = 0.75, *α*_t12_ = 0.92, *α*_t2_ = 0.79).

##### 3.2.1.6 Collective efficacy beliefs

Based on van Zomeren et al. (2013), I assessed collective efficacy beliefs towards precycling with two items. Participants rated the statements (“I believe that we, as members of the HomeLabs, can drive precycling” and “I believe that we, as members of the HomeLabs, can contribute to solving the environmental crisis through joint actions”) on a 7-point Likert scale (from 1 = “totally disagree” to 7 = “totally agree”). The analysis is based on the individual mean score (*α*_t0_ = 0.74, *α*_t1_ = 0.75, *α*_t12_ = 0.92, *α*_t2_ = 0.79).

##### 3.2.1.7 Appraisal of the crisis

The appraisal of the packaging waste as a problem was measured with seven of the original eight items adapted from BMUB and UBA (2017) and Heidbreder et al. (2020) using a five-point Likert (from 1–“no problem” to 5–“very big problem”). The participants rated their perception of different environmental problems related to packaging waste (e.g., energy and resource consumption, packaging waste in the ocean). The analysis is based on the individual mean score (*α*_t0_ = 0.63, *α*_t1_ = 0.63, *α*_t12_ = 0.59, *α*_t2_ = 0.61).

##### 3.2.1.8 Sufficiency attitude

The attitude towards a sufficiency-oriented lifestyle was measured with a six-item scale based on Henn (2013) and Verfuerth et al. (2019): accordingly, the participants were asked to rate their agreement towards six statements about the waste of resources and a frugal lifestyle on a six-point Likert scale (1 = strongly disagree, 6 = strongly agree). The analysis is based on the individual mean score (*α*_t0_ = 0.67, *α*_t1_ = 0.67, *α*_t12_ = 0.76, *α*_t2_ = 0.68).

### 3.3 Data analysis

I performed the data analysis with R Statistics version 4.0.5. To test the hypotheses, I conducted step-wise multi-level regression analyses with random intercept. This procedure results from the study design with up to four repeated measurements and more than one person per household, hence, interdependent and hierarchically structured data. Thus, each intercept-only model includes the persons (*n* = 126) nested in households (*n* = 94). I calculated the intra-class-correlation (ICC) to measure the strength of dependence of the data. In all models presented in this paper, the ICC was higher than 0.1 which supports my account for using multi-level models (see Vajargah and Nikbakht, 2015). For each research question, I specified the intercept-only model by defining the dependent and independent variables of interest. The presented models are specified without random slope variance. I did not define covariance structure because the repeated measure has been considered by including the time variable as a predictor. In the stepwise regression, the measurement at the respective measurement time was used for the independent variables ingroup identity, norms, collective efficacy beliefs, and goal. This means that the effect of these variables on behavior at the respective time point was analyzed. I used the difference in the −2* Log-Likelihood values of two models to calculate a chi-square statistic and to test whether the model has improved significantly by adding the respective predictors. In general, for testing multilevel models, it is desirable to have many clusters at the top level (Snijders, 2005), in this case the households. In terms of accuracy and power, a large number of households is more important than a large number of individuals per households (Hox et al., 2018). More specifically, simulation studies have shown that using less than 50 clusters in complex multilevel models (e.g., testing for indirect effects) is problematic when using Maximum Likelihood (ML) estimation (Hox et al., 2014). Because the sample size at the top level in the present study is relatively large (92 households) and the models are of low complexity in terms of random effects (only one random intercept, no indirect effects), it is appropriate to use a multilevel model with ML estimation. In order to obtain information about the power of a multilevel model, power can also be considered as a consequence of the standard error of estimation (Snijders, 2005).

I included the post and follow-up data of participants according to the following criteria: Participants assigned to intervention group IG1 or IG2 were included in the analysis if they had at least participated in the kick-off webinar – either synchronically or watched the recording. All participants in the waiting control group were included. This proceeding resulted in a sample of 126 participants with at least one completed time of measurement. The regression analyses are based on this sample of 126 participants.

The data of the waiting control group (WCG) were restructured. Figure 1 visualizes the restructuring procedure of the data. Figure 1 shows that the WCG had four measurement points, which were than divided into two separate periods: a control period and an intervention period (webinar). The control period was then defined by the data collected at t0 and t1 during the waiting period without intervention (representing the control group). The data t1 and t1b were used to simulate another intervention group, representing the effect of the webinars. For this “new” intervention group four the follow-up data t2 was also included. This allowed the effects of the waiting period and the webinar intervention to be modeled separately. Instead of the control group, group four was used as the reference group so that the long-term effects of the other interventions could also be modeled and the effect of the webinars could be controlled for.

Through this restructuring, the waiting control group participants were represented twice in the analysis, once in the control group, and once in the “new” intervention group. Thus, the models reported in the result section 3.2 consider four group conditions. Multilevel models represent an appropriate approach to analyzing this kind of dependent data structure.

For all models reported, homoscedasticity and normal distribution of residuals are approximately given. No extreme outliers were detected in the residuals. The normal distribution of random constants is approximately given. To check for collinearity, I used the squared scaled GVIF. All squared scaled GVIF are squared scaled GVIF were below five, mostly below 4, so there was no need for action. All metric, independent variables were grand mean centered. As effect size, I report Cohen’s f^2^ (Cohen, 1988), calculated based on Pseudo-R-squared for Generalized Mixed-Effect models (conditional R-squared). It is important to note that there are no standard guidelines for what is an appropriate effect size for complex multilevel models. In this study, Cohen’s f^2^ (Cohen, 1988) is used, but all effect sizes given here should be understood as an approximate guide only.

## 4 Results

### 4.1 Explorative factor analysis

The maximum likelihood (ML) method with orthogonal varimax rotation was applied to prove that the items to measure reuse behavior can be considered as a single-factor scale. Verifying the suitability of the sample for the analysis with the Kaiser-Mayer-Olkin method resulted in KMO = 0.64, evaluated as mediocre according to Kaiser (1974). All individual items were > 0.55, confirming that the sample can be used for the analysis. Bartlett’s test of sphericity proved the correlations as being sufficiently large for ML (χ^2^ (15) = 67.69, *p* < 0.001). Then the Velicer MAP test was run and achieved a minimum of 0.05 with 1 factor. Also, an analysis was run to obtain the eigenvalues for each component resulting in 2 components having eigenvalues over Kaiser’s criterion of 1. Based on thematic considerations and suitability, I decided to consider one factor in the final analysis. Table 3 displays the factor loadings after varimax rotation.

**Table 3:** Results from the explorative factor analysis of the reuse behavior items.

	Varimax rotated factor loadings
Item	Reuse behavior
I use my grocery bags multiple times.	0.29
I reuse food packaging for the same purpose (e.g., paper bags for bread).	0.54
I reuse food packaging for other purposes (e.g., tetra pack for crafting).	0.55
I use reusable eating utensils when I am on the road (e.g., travel coffee mug, water bottle, reusable containers).	0.21
To bring my shopping home, I use my own bag, not a store-provided one.	0.28
I use my own containers to buy unpackaged food products.	0.65
Eigenvalues	1.94
% of variance	0.20
*α*	0.55

*N* = 125.

### 4.2 Multilevel analyses

In this section, I report the results of the hypothesis tests. The final regression models include the hypothesized social identity variables, and the control variables gender, age, household size and education. All models include time as a repeated measure, with t0 as a reference, coded with zero. To analyze the effects of the different intervention conditions the intervention group four (IG4, representing the delayed intervention of the waiting control group) was set as reference group, coded with zero. Correlations of all main model components at the baseline are shown in Tables 4–6 present means and standard deviations for each condition and time point of measure separately, before and after restructuring the data of the WCG.

**Table 4:** Correlation table for measures in the analyzed sample, before (*N* = 132) and after (*n* = 126) restructuring the data.

	1	2	3	4	5	6	7	8	9
*Before*									
1 Precycling behavior	–								
2 Reuse behavior	0.45***	–							
3 Ingroup identification	0.27***	0.16**	–						
4 Descriptive norms	0.16**	0.12*	0.13*	–					
5 Injunctive norms	0.06	0.02	0.13*	0.48***	–				
6 Collective efficacy beliefs	0.28***	0.14*	0.31***	0.08	0.13*	–			
7 Sufficiency attitude	0.37***	0.2***	0.23***	−0.01	−0.04	0.09	–		
8 Appraisal of crisis	0.26***	0.25***	0.05	−0.04	0.08	0.18**	0.26***	–	
9 Goal	0.5***	0.26***	0.28***	0.11	0.05	0.25***	0.24***	0.23***	–
*After*									
1 Precycling behavior	-–								
2 Reuse behavior	0.4***	–							
3 Ingroup identification	0.27***	0.19***	–						
4 Descriptive norms	0.16**	0.15**	0.17**	–					
5 Injunctive norms	0.07	0.07	0.21***	0.49***	-–				
6 Collective efficacy beliefs	0.21***	0.1	0.32***	0.09	0.14*	-–			
7 Sufficiency attitude	0.34***	0.16**	0.21***	−0.03	−0.05	0.07	–		
8 Appraisal of crisis	0.18**	0.2***	0.02	−0.08	0.07	0.17**	0.27***	–-	
9 Goal	0.47***	0.37***	0.28***	0.11	0.08	0.21***	0.2***	0.17**	–-

*p* < 0.10. *p < 0.05. **p < 0.01. ***p < 0.001.

**Table 5:** Means and standard deviations separately for the different groups and each time of, before restructuring the data (*N* = 132).

		Intervention group 1 (IG1) (n_0_ = 44, n_1_ = 31, n_1b_ = 0, n_2_ = 28)		Intervention group 2 (IG2) (n_0_ = 39, n_1_ = 26, n_1b_ = 0, n_2_ = 29)		Waiting control group (WCG) (n_0_ = 43, n_1_ = 33, n_1b_ = 24, n_2_ = 28)	
	Time	*M*	*SD*	*M*	*SD*	*M*	*SD*
Precycling behavior	t0	4.54	1.43	4.98	1.10	4.93	1.04
	t1	4.83	1.34	5.28	1.00	5.00	0.92
	t1b	–	–	–	–	5.42	0.84
	t2	4.36	1.55	5.04	1.07	5.10	1.19
Reuse behavior	t0	4.02	0.47	3.82	0.53	3.91	0.48
	t1	3.95	0.61	4.01	0.41	3.79	0.43
	t1b	–	–	–	–	3.78	0.47
	t2	3.92	0.56	3.86	0.54	3.83	0.38
Ingroup identification	t0	2.58	0.71	2.50	0.72	2.73	0.67
	t1	2.65	0.61	3.09	0.83	2.54	0.78
	t1b	–	–	–	–	2.64	0.72
	t2	2.14	0.77	2.93	0.68	2.66	0.71
Descriptive norms	t0	3.66	0.64	3.85	0.63	3.86	0.68
	t1	3.84	0.58	4.12	0.65	4.00	0.56
	t1b	–	–	–	–	3.92	0.58
	t2	4.14	0.59	3.97	0.78	4.00	0.61
Injunctive norms	t0	6.40	0.74	6.26	0.99	6.48	0.73
	t1	6.61	0.54	6.65	0.52	6.42	0.82
	t1b	–	–	–	–	6.58	0.70
	t2	6.59	0.49	6.64	0.58	6.52	0.74
Collective efficacy beliefs	t0	5.38	1.20	4.91	1.44	5.35	1.28
	t1	5.45	1.39	4.87	1.25	5.23	1.23
	t1b	–	–	–	–	4.75	1.70
	t2	4.68	1.74	4.72	1.51	4.79	1.47
Sufficiency attitude	t0	4.52	0.77	4.48	0.83	4.60	0.82
	t1	4.55	0.77	4.49	076	4.66	0.87
	t1b	–	–	–	–	4.70	0.83
	t2	4.52	0.81	4.44	0.81	4.73	0.82
Appraisal of crisis	t0	4.66	0.39	4.66	0.30	4.65	0.40
	t1	4.70	0.39	4.66	0.32	4.66	0.33
	t1b	–	–	–	–	4.61	0.40
	t2	4.71	0.40	4.63	0.30	4.62	0.41
Goal	t0	4.36	0.92	4.69	0.66	4.51	0.70
	t1	3.97	0.87	4.35	0.89	3.91	0.95
	t1b	–	–	–	–	4.17	0.92
	t2	3.81	1.11	4.10	0.77	4.29	0.71

t0 = prae; t1 = post (IG1 and IG2); t12 = post (WCG); t2 = follow up. Sufficiency attitude and appraisal of crisis were only measured at t0.

**Table 6:** Means and standard deviations separately for the different groups and times of measure in the analyzed sample, after restructuring the data (*n* = 126).

		Intervention group 1 (IG1) (n_0_ = 43, n_1_ = 19, n_2_ = 16)		Intervention group 2 (IG2) (n_0_ = 39, n_1_ = 21, n_2_ = 20)		Control group (CG) (n_0_ = 43, n_1_ = 32)		Intervention group 4 (IG4) (n0 = 32, n1 = 24, n2 = 18)	
	Time	*M*	*SD*	*M*	*SD*	*M*	*SD*	*M*	*SD*
Precycling behavior	t0	4.52	1.44	4.98	1.10	4.93	1.04	5.03	0.92
	t1	4.87	1.21	5.22	0.92	5.03	0.92	5.42	0.84
	t2	4.09	1.43	5.13	1.15	–	–	5.43	0.91
Reuse behavior	t0	4.02	0.47	3.82	0.53	3.91	0.48	3.80	0.43
	t1	3.91	0.66	4.06	0.37	3.80	0.43	3.78	0.47
	t2	3.83	0.55	4.04	0.41	–	–	3.83	0.35
Ingroup identification	t0	2.56	0.70	2.50	0.72	2.73	0.67	2.55	0.78
	t1	2.93	0.40	3.11	0.70	2.55	0.78	2.64	0.72
	t2	2.39	0.76	2.97	0.65	–	–	2.91	0.66
Descriptive norms	t0	3.67	0.64	3.85	0.63	3.86	0.68	4.00	0.57
	t1	3.84	0.50	4.10	0.62	4.00	0.57	3.92	0.58
	t2	3.94	0.57	3.85	0.81	–	–	4.11	0.68
Injunctive norms	t0	6.40	0.74	6.26	0.99	6.48	0.73	6.41	0.83
	t1	6.58	0.63	6.64	0.55	6.41	0.83	6.58	0.70
	t2	6.50	0.55	6.47	0.64	–	–	6.44	0.84
Collective efficacy beliefs	t0	5.38	1.21	4.91	1.44	5.35	1.28	5.20	1.24
	t1	5.79	1.07	4.76	1.35	5.20	1.24	4.75	1.70
	t2	4.84	1.66	4.60	1.61	–	–	4.78	1.49
Sufficiency attitude	t0	4.52	0.78	4.48	0.83	4.60	0.82	4.66	0.87
	t1	4.64	0.63	4.56	0.70	4.66	0.87	4.70	0.83
	t2	4.64	0.66	4.54	0.72	–	–	4.72	0.80
Appraisal of crisis	t0	4.65	0.39	4.66	0.30	4.65	0.40	4.66	0.33
	t1	4.73	0.34	4.61	0.32	4.66	0.87	4.61	0.40
	t2	4.71	0.35	4.61	0.33	–	–	4.60	0.39
Precycling goal	t0	4.35	0.92	4.69	0.66	4.51	0.70	3.91	0.96
	t1	3.98	0.94	4.29	0.90	3.91	0.96	4.17	0.92
	t2	3.75	1.13	4.20	0.83	–	–	4.44	0.51

T0 = pre; t1 = post; t2 = follow up. Sufficiency attitude and appraisal of crisis were only measured at t0. The control group (CG) has no data at t2 because of the data restructuring.

#### 4.2.1 Pro-environmental response

The results of the pre-post intervention effects on precycling and on reuse behavior as a subset of precycling are presented in this text section and in Tables 7, 8. The results, including the follow-up data, are presented in Tables 9, 10.

**Table 7:** Step-wise mixed-model regression of precycling behavior as interaction of time (t0/t1) and the intervention groups, including social identity and control variables.

	Precycling behavior															
Predictor	Model 0		Model 1		Model 2		Model 3		Model 4		Model 5		Model 6			
	*b (SE)*	*p*	*b (SE)*	*p*	*b (SE)*	*p*	*b (SE)*	*p*	*b (SE)*	*p*	*b (SE)*	*p*	*b (SE)*	95% CI	*p*	*f* ^2^
*Fixed effects*																
Intercept	4.90 (0.11)	<0.001***	4.86 (0.11)	<0.001***	4.98 (0.19)	<0.001***	4.89 (0.20)	<0.001***	5.00 (0.19)	<0.001***	5.05 (0.17)	<0.001***	4.54 (0.34)	3.87; 5.22	<0.001***	
Time [1]			0.14 (0.06)	0.033*	0.14 (0.06)	0.030*	0.11 (0.12)	0.396	0.14 (0.13)	0.270	0.13 (0.13)	0.324	0.14 (0.13)	−0.12; 0.41	0.282	0.02
Intervention group 1					−0.35 (0.27)	0.202	−0.38 (0.28)	0.169	−0.42 (0.27)	0.116	−0.50 (0.23)	0.031*	−0.55 (0.22)	−0.98; −0.12	0.012*	0.01
Intervention group 2					0.04 (0.27)	0.871	0.02 (0.28)	0.937	0.04 (0.27)	0.871	−0.10 (0.23)	0.681	−0.13 (0.22)	−0.57; 0.31	0.567	
Control group 3					−0.08 (0.08)	0.315	−0.06 (0.11)	0.607	−0.11 (0.11)	0.348	−0.20 (0.12)	0.100	−0.17 (0.12)	−0.42; 0.07	0.166	
Time[1]: intervention group 1							0.15 (0.19)	0.420	0.04 (0.20)	0.850	0.11 (0.21)	0.600	0.09 (0.21)	−0.32; 0.50	0.663	0.01
Time[1]: intervention group 2							0.09 (0.18)	0.628	0.01 (0.19)	0.964	0.10 (0.20)	0.612	0.08 (0.20)	−0.31; 0.47	0.679	
Time[1]: control group 3							−0.05 (0.17)	0.767	−0.04 (0.17)	0.836	0.07 (0.19)	0.704	0.03 (0.19)	−0.34; 0.40	0.881	
Ingroup identification									0.13 (0.09)	0.125	0.07 (0.08)	0.390	0.03 (0.09)	−0.14; 0.20	0.743	<0.01
Descriptive norms									0.01 (0.08)	0.927	0.05 (0.08)	0.551	0.07 (0.08)	−0.10; 0.24	0.414	<0.01
Injunctive norms									−0.02 (0.07)	0.750	−0.04 (0.07)	0.556	−0.05 (0.07)	−0.19; 0.09	0.522	<0.01
Collective efficacy beliefs									0.12 (0.05)	0.009**	0.12 (0.04)	0.009**	0.12 (0.04)	0.03; 0.21	0.006**	<0.01
Sufficiency attitude											0.38 (0.10)	<0.001***	0.36 (0.10)	0.14; 0.57	<0.001***	<0.01
Appraisal of crisis											0.50 (0.22)	0.026*	0.57 (0.22)	0.14; 1.02	0.011*	0.04
Goal											0.18 (0.06)	0.004**	0.18 (0.06)	0.05; 0.32	0.004**	<0.01
Gender (female)													0.13 (0.17)	−0.21; 0.49	0.434	<0.01
Age													0.00 (0.01)	−0.01; 0.01	0.897	<0.01
Household size													−0.17 (0.07)	−0.31; −0.03	0.015*	0.02
Education (university degree)													0.25 (0.17)	−0.08; 0.59	0.131	<0.01
*Random effects*																
Level 1 intercept σ^2^	0.59		0.59		0.60		0.61		0.52		0.38		0.45			
Level 2 intercept σ^2^	0.56		0.55		0.51		0.50		0.47		0.28		0.14			
Goodness of fit																
-2LL	609.81		605.23		601.94		600.42		588.57		554.56		546.66			
∆χ2			4.58*		3.29		1.52		11.85*		34.01***		7.90°			
∆*df*			1		3		3		4		3		4			

For all models *n* of level 1 (persons) = 126. *N* of level 2 (households) = 92. Intervention group 4 is the reference group. Time [0] represents the pre measure and is the reference group for time of measure, time [1] represents the post-measure. °*p* < 0.10. *p < 0.05. **p < 0.01. ***p < 0.001.

**Table 8:** Step-wise mixed-model regression of reuse behavior as interaction of time (t0/t1) and the intervention groups, including social identity and control variables.

	Reuse behavior															
Predictor	Model 0		Model 1		Model 2		Model 3		Model 4		Model 5		Model 6			
	*b (SE)*	*p*	*b (SE)*	*p*	*b (SE)*	*p*	*b (SE)*	*p*	*b (SE)*	*p*	*b (SE)*	*p*	*b (SE)*	95% CI	*p*	*f^2^*
Fixed effects																
Intercept	3.91 (0.04)	<0.001***	3.92 (0.04)	<0.001***	3.79 (0.08)	<0.001***	3.80 (0.08)	<0.001***	3.80 (0.08)	<0.001***	3.82 (0.07)	<0.001***	3.66 (0.10)	3.45; 3.86	<0.001***	
Time [1]			−0.03 (0.03)	0.306	−0.03 (0.03)	0.274	−0.08 (0.06)	0.174	−0.07 (0.06)	0.265	−0.08 (0.06)	0.197	−0.08 (0.06)	−0.20; 0.05	0.223	0.08
Intervention group 1					0.23 (0.11)	0.038*	0.22 (0.11)	0.045*	0.23 (0.11)	0.035*	0.20 (0.10)	0.057°	0.18 (0.10)	−0.01; 0.37	0.064°	0.09
Intervention group 2					0.10 (0.11)	0.345	0.03 (0.11)	0.789	0.04 (0.11)	0.705	−0.02 (0.11)	0.846	−0.04 (0.10)	−0.24; 0.16	0.689	
Control group 3					0.09 (0.04)	0.029*	0.11 (0.05)	0.049*	0.10 (0.05)	0.063°	0.06 (0.06)	0.315	0.07 (0.06)	−0.04; 0.19	0.206	
Time[1]: intervention group 1							−0.00 (0.09)	0.997	−0.05 (0.10)	0.617	−0.01 (0.10)	0.944	−0.01 (0.10)	−0.21; 0.18	0.886	0.09
Time[1]: intervention group 2							0.25 (0.09)	0.005**	0.22 (0.09)	0.018*	0.27 (0.09)	0.004**	0.27 (0.09)	0.09; 0.46	0.004**	
Time[1]: control group 3							−0.02 (0.08)	0.783	−0.03 (0.08)	0.703	0.02 (0.09)	0.798	0.00 (0.09)	−0.17; 0.18	0.974	
Ingroup identification									0.03 (0.04)	0.428	0.00 (0.04)	0.932	−0.02 (0.04)	−0.11; 0.06	0.549	<0.01
Descriptive norms									0.05 (0.04)	0.206	0.05 (0.04)	0.184	0.06 (0.04)	−0.02; 0.14	0.123	<0.01
Injunctive norms									0.00 (0.03)	0.973	−0.00 (0.03)	0.883	−0.00 (0.03)	−0.17; 0.06	0.944	<0.01
Collective efficacy beliefs									0.02 (0.02)	0.355	0.02 (0.02)	0.455	0.01 (0.02)	−0.03; 0.05	0.513	0.01
Sufficiency attitude											0.08 (0.05)	0.116	0.07 (0.05)	−0.03; 0.16	0.181	<0.01
Appraisal of crisis											0.30 (0.11)	0.005**	0.33 (0.11)	0.12; 0.54	0.002**	<0.01
Goal											0.08 (0.03)	0.009**	0.08 (0.03)	0.02; 0.14	0.010*	<0.01
Gender (female)													0.18 (0.08)	0.02; 0.34	0.028*	<0.01
Age													−0.00 (0.00)	−0.01; 0.00	0.516	<0.01
Household size													−0.04 (0.03)	−0.10; 0.03	0.241	<0.01
Education [university degree]													0.06 (0.08)	−0.10; 0.22	0.442	<0.01
Random effects																
Level 1 intercept σ^2^	0.16		0.16		0.16		0.16		0.16		0.12		0.13			
Level 2 intercept σ^2^	0.03		0.03		0.03		0.3		0.02		0.02		0.00			
Goodness of fit																
-2LL	226.29		225.24		217.67		205.25		200.85		177.73		170.84			
∆χ2			1.74		7.57°		12.42**		4.39		23.13***		6.88			
∆*df*			2		3		3		4		3		4			

For all models *n* of level 1 (persons) = 126. *N* of level 2 (households) = 92. Intervention group 4 is the reference group. Time [0] represents the pre measure and is the reference group for time of measure, time [1] represents the post-measure. °*p* < 0.10. *p < 0.05. **p < 0.01. ***p < 0.001.

**Table 9:** Step-wise mixed-model regression of precycling behavior as interaction of time (t0/ t1/t2) and the intervention groups, including social identity and control variables.

	Precycling behavior															
Predictor	Model 0		Model 1		Model 2		Model 3		Model 4		Model 5		Model 6			
	*b (SE)*	*p*	*b (SE)*	*p*	*b (SE)*	*p*	*b (SE)*	*p*	*b (SE)*	*p*	*b (SE)*	*p*	*b (SE)*	95% CI	*p*	*f* ^2^
*Fixed effects*																
Intercept	4.89 (0.11)	<0.001***	4.86 (0.11)	<0.001***	4.99 (0.20)	<0.001***	4.97 (0.20)	<0.001***	4.99 (0.20)	<0.001***	5.05 (0.17)	<0.001***	4.82 (0.23)	4.36; 5.27	<0.001***	
Time [t1]			0.13 (0.07)	0.065°	0.13 (0.07)	0.059°	0.13 (0.14)	0.354	0.17 (0.14)	0.240	0.15 (0.14)	0.316	0.15 (0.14)	−0.13; 0.44	0.289	0.01
Time [t2]			−0.01 (0.09)	0.902	−0.03 (0.09)	0.744	0.03 (0.15)	0.850	0.04 (0.16)	0.791	−0.02 (0.16)	0.913	−0.01 (0.16)	−0.32; 0.31	0.970	
Intervention group 1					−0.37 (0.27)	0.169	−0.36 (0.28)	0.203	−0.40 (0.27)	0.142	−0.49 (0.23)	0.039*	−0.53 (0.22)	−0.97; −0.08	0.020*	<0.01
Intervention group 2					0.04 (0.27)	0.885	0.03 (0.28)	0.918	0.04 (0.27)	0.881	−0.12 (0.24)	0.624	−0.15 (0.23)	−0.60; 0.31	0.530	
Control group 3					−0.10 (0.09)	0.266	−0.05 (0.12)	0.712	−0.09 (0.13)	0.459	−0.20 (0.13)	0.137	−0.17 (0.13)	−0.43; 0.09	0.207	
Time[t1]: intervention group 1							0.10 (0.21)	0.637	0.00 (0.22)	0.997	0.09 (0.22)	0.673	0.08 (0.22)	−0.36; 0.51	0.726	<0.01
Time[t2]: intervention group 1							−0.17 (0.23)	0.456	−0.10 (0.24)	0.684	−0.04 (0.24)	0.882	−0.07 (0.24)	−0.54; 0.41	0.782	
Time[t1]: intervention group 2							0.06 (0.21)	0.759	−0.02 (0.21)	0.910	0.08 (0.21)	706	0.06 (0.21)	−0.36; 0.48	0.774	
Time[t2]: intervention group 2							0.03 (0.22)	0.876	0.03 (0.22)	0.904	0.18 (0.22)	0.414	0.15 (0.22)	−0.29; 0.59	0.509	
Time[t1]: control group 3							−0.09 (0.19)	0.652	−0.08 (0.20)	0.697	0.05 (0.20)	0.804	0.01 (0.20)	−0.38; 0.41	0.950	
Ingroup identification									0.14 (0.08)	0.079°	0.09 (0.08)	0.232	0.06 (0.08)	−0.10; 0.22	0.460	<0.01
Descriptive norms									0.02 (0.07)	0.761	0.06 (0.07)	0.442	0.06 (0.07)	−0.08; 0.21	0.386	<0.01
Injunctive norms									−0.05 (0.07)	0.430	−0.06 (0.07)	0.344	−0.06 (0.07)	−0.20; 0.07	0.338	<0.01
Collective efficacy beliefs									0.11 (0.04)	0.010**	0.09 (0.04)	0.032*	0.09 (0.04)	0.01; 0.17	0.026*	<0.01
Sufficiency attitude											0.37 (0.10)	<0.001***	0.35 (0.10)	0.15; 0.56	<0.001***	<0.01
Appraisal of crisis											0.52 (0.21)	0.018*	0.57 (0.22)	0.15; 1.01	0.009**	0.03
Goal											0.19 (0.05)	<0.001***	0.19 (0.06)	0.08; 0.30	<0.001***	<0.01
Gender (female)													0.13 (0.16)	−0.20; 0.47	0.433	<0.01
Age													0.00 (0.01)	−0.01; 0.01	0.945	<0.01
Household size													−0.14 (0.07)	−0.28; 0.00	0.051°	0.03
Education (university degree)													0.22 (0.17)	−0.10; 0.56	0.178	<0.01
*Random effects*																
Level 1 Intercept σ^2^	0.51		0.52		0.52		0.52		0.43		0.31		0.35			
Level 2 Intercept σ^2^	0.62		0.61		0.56		0.55		0.51		0.26		0.23			
Goodness of fit																
-2LL	738.51		734.30		730.51		728.07		715.59		675.92		670.19			
∆χ2			4.20		3.80		2.44		12.48*		39.67***		5.73			
∆*df*			2		3		5		4		3		4			

For all models *n* of level 1 (persons) = 126. *N* of level 2 (households) = 92. Intervention group 4 is the reference group. Time [0] represents the pre measure and is the reference group for time of measure, time [1] represents the post measure, and time [2] the follow-up. °*p* < 0.10. *p < 0.05. **p < 0.01. ***p < 0.001.

**Table 10:** Step-wise mixed-model regression of reuse behavior as interaction of time (t0/ t1/t2) and the intervention groups, including social identity and control variables.

	Reuse behavior															
Predictor	Model 0		Model 1		Model 2		Model 3		Model 4		Model 5		Model 6			
	*b (SE)*	*p*	*b (SE)*	*p*	*b (SE)*	*p*	*b (SE)*	*p*	*b (SE)*	*p*	*b (SE)*	*p*	*b (SE)*	95% CI	*p*	*f* ^2^
*Fixed effects*																
Intercept	3.90 (0.04)	<0.001***	3.92 (0.04)	<0.001***	3.79 (0.08)	<0.001***	3.80 (0.08)	<0.001***	3.80 (0.08)	<0.001***	3.82 (0.07)	<0.001***	3.67 (0.10)	3.47; 3.87	<0.001***	
Time [1]			−0.03 (0.04)	0.348	−0.04 (0.04)	0.329	−0.07 (0.07)	0.316	−0.06 (0.07)	0.403	−0.08 (0.07)	0.295	−0.07 (0.07)	−0.21; 0.07	0.312	0.06
Time [2]			−0.04 (0.05)	0.373	−0.03 (0.05)	0.565	−0.02 (0.08)	0.778	−0.02 (0.08)	0.771	−0.05 (0.08)	0.497	−0.05 (0.08)	−0.021; 0.11	0.538	
Intervention group 1					0.20 (0.10)	0.058°	0.23 (0.11)	0.038	0.24 (0.11)	0.030*	0.20 (0.10)	0.049*	0.19 (0.10)	−0.01; 0.38	0.057°	0.06
Intervention group 2					0.12 (0.11)	0.253	0.04 (0.11)	0.747	0.05 (0.11)	0.678	−0.02 (0.10)	0.812	−0.04 (0.10)	−0.24; 0.16	0.686	
Control group 3					0.09 (0.05)	0.064°	0.11 (0.06)	0.072°	0.11 (0.06)	0.095°	0.06 (0.06)	0.365	0.07 (0.06)	−0.06; 0.20	0.268	
Time[1]: intervention group 1							−0.02 (0.11)	0.849	−0.07 (0.11)	0.545	−0.02 (0.11)	0.891	−0.02 (0.11)	−0.24; 0.19	0.849	0.05
Time[2]: intervention group 1							−0.18 (0.11)	0.122	−0.18 (0.12)	0.137	−0.13 (0.12)	0.279	−0.15 (0.12)	−0.38; 0.09	0.222	
Time[1]: intervention group 2							0.25 (0.10)	0.018*	0.20 (0.10)	0.053°	0.26 (0.11)	0.013*	0.26 (0.11)	0.06; 0.47	0.013*	
Time[2]: intervention group 2							0.19 (0.11)	0.080°	0.19 (0.11)	0.088°	0.27 (0.11)	0.018*	0.26 (0.11)	0.04; 0.48	0.022*	
Time[1]: control group 3							−0.01 (0.09)	0.667	−0.05 (0.10)	0.642	0.02 (0.10)	0.868	0.00 (0.10)	−0.20; 0.20	0.996	
Ingroup identification									0.05 (0.04)	0.185	0.03 (0.04)	0.503	0.00 (0.04)	−0.08; 0.08	0.985	<0.01
Descriptive norms									0.05 (0.04)	0.158	0.06 (0.04)	0.109	0.06 (0.04)	−0.01; 0.13	0.089°	<0.01
Injunctive norms									−0.00 (0.03)	0.891	−0.01 (0.03)	0.778	−0.01 (0.03)	−0.07; 0.06	0.861	<0.01
Collective efficacy beliefs									0.02 (0.02)	0.405	0.01 (0.02)	0.784	0.00 (0.02)	−0.03; 0.04	0.815	<0.01
Sufficiency attitude											0.08 (0.05)	0.076°	0.07 (0.05)	−0.02; 0.17	0.130	<0.01
Appraisal of crisis											0.25 (0.10)	0.018*	0.27 (0.10)	0.07; 0.47	0.009**	<0.01
Goal											0.09 (0.03)	0.001**	0.09 (0.03)	0.03; 0.14	0.002**	<0.01
Gender (female)													0.16 (0.08)	0.01; 0.32	0.039*	<0.01
Age													−0.00 (0.00)	−0.01; 0.00	0.648	<0.01
Household size													−0.03 (0.03)	−0.09; 0.03	0.328	<0.01
Education (university degree)													0.06 (0.08)	−0.10; 0.21	0.453	<0.01
*Random effects*																
Level 1 Intercept σ^2^	0.13		0.13		0.13		0.14		0.13		0.11		0.11			
Level 2 Intercept σ^2^	0.03		0.03		0.03		0.03		0.02		0.01		0.00			
Goodness of fit																
-2LL	281.10		279.88		274.19		258.61		252.39		227.83		222.03			
∆χ2			1.22		5.69		15.59**		6.22		24.56***		5.80			
∆*df*			2		3		5		4		3		4			

For all models *n* of level 1 (persons) = 126. *N* of level 2 (households) = 92. Intervention group 4 is the reference group. Time [0] represents the pre measure and is the reference group for time of measure, time [1] represents the post measure, and time [2] the follow-up. °*p* < 0.10. *p < 0.05. **p < 0.01. ***p < 0.001.

With regard to precycling behavior, the results do not confirm hypothesis 1.1. The intervention does not result in significantly higher precycling behavior when comparing the different group conditions. However, when using a very basic model (see Table 7, model 1) where only the main effect of time is considered as predictor of precycling, precycling behavior increased significantly after the intervention (*b* = 0.14, *p* = 0.033). Nevertheless, the significance of the increased precycling behavior between baseline and post-intervention disappears as soon as the interaction with the intervention groups is added.

With regard to reuse behavior, the results partly support hypothesis 1.1: Compared to the other groups, the intervention results in significantly higher reuse behavior in the second intervention group (*b* = 0.27 [0.09, 0.46], *p* = 0.004). The overall the interaction effect of time and group appears to be small (*f^2^* = 0.09).

Following the SIMPEA, I hypothesized that the social identity variables of ingroup identification, ingroup norms, and collective efficacy beliefs would predict precycling behavior. These assumptions are only partially supported by the data: The results do not support hypothesis 1.2. Ingroup identification does not significantly predict precycling behavior (*b* = 0.03 [−0.14, 0.20], *p* = 0.743). Neither does it predict reuse behavior (*b* = −0.02 [−0.11, 0.06], *p* = 0.549). The results do not support hypothesis 1.3. Descriptive (*b* = 0.07 [−0.10, 0.24], *p* = 0.414) and injunctive ingroup norms (*b* = −0.05 [−0.19, 0.09], *p* = 0.522) do not significantly predict precycling behavior. Neither do descriptive (*b* = 0.06 [0.02, 0.14], *p* = 0.123) and injunctive ingroup norms (*b* = −0.00 [−0.17, 0.06], *p* = 0.944) predict reuse behavior. Last but not least, the results support Hypothesis 1.4. Collective efficacy beliefs towards the participants of the HomeLab-Community significantly and positively predict precycling behavior (*b* = 0.12 [0.03, 0.21], *p* = 0.006), but not reuse behavior (*b* = 0.01 [−0.03, 0.05], *p* = 0.513). However, the positive influence of collective efficacy beliefs seems not relevant in terms of effect sizes (*f^2^* < 0.01).

#### 4.2.2 Ingroup identification

The analysis of the pre-post-intervention effects on ingroup identification as formulated in hypothesis 2.1. is presented in Table 11. The results, which include the follow-up data, are shown in Table 12. The results partly confirm Hypothesis 2.1. Compared to the reference group four, the ingroup identification changed in group two and the control group. Participants in group two identified significantly stronger with the HomeLab community after the intervention (*b* = 0.33 [0.01, 0.065], *p* = 0.042) whereas the control group identified significantly less after the intervention period (*b*  = −0.37 [−0.66, −0.08], *p* = 0.042). Ingroup identification in intervention group one did not change significantly between t0 and t1. Overall, this interaction of time and group shows a medium effect (*f^2^*  = 0.16).

**Table 11:** Mixed-model regression of ingroup identification as interaction of time (t0/t1) and the intervention groups, including control variables.

	Ingroup identification											
Predictor	Model 0		Model 1		Model 2		Model 3		Model 4			
	*b (SE)*	*p*	*b (SE)*	*p*	*b (SE)*	*p*	*b (SE)*	*p*	*b (SE)*	95% CI	*p*	*f* ^2^
Fixed effects												
Intercept	2.66 (0.06)	<0.001***	2.63 (0.07)	<0.001***	2.54 (0.12)	<0.001***	2.50 (0.12)	<0.001***	2.00 (0.23)	1.54; 2.46	<0.001***	
Time [1]			0.10 (0.06)	0.100	0.10 (0.06)	0.106	0.11 (0.11)	0.323	0.11 (0.11)	−0.11; 0.32	0.338	0.15
Intervention group 1					0.11 (0.16)	0.489	0.11 (0.17)	0.515	0.05 (0.16)	−0.26; 0.35	0.766	0.17
Intervention group 2					0.11 (0.16)	0.483	0.04 (0.17)	0.833	−0.02 (0.16)	−0.33; 0.29	0.912	
Control group 3					0.08 (0.08)	0.289	0.25 (0.10)	0.010*	0.27 (0.10)	0.08; 0.45	0.066**	
Time[1]: intervention group 1							0.12 (0.17)	0.486	0.10 (0.16)	−0.22; 0.43	0.541	0.16
Time[1]: intervention group 2							0.35 (0.16)	0.030*	0.33 (0.16)	0.01; 0.65	0.042*	
Time[1]: control group 3							−0.36 (0.15)	0.016*	−0.37 (0.15)	−0.66; −0.08	0.013*	
Gender [female]									0.46 (0.11)	0.24; 0.67	<0.001***	<0.01
Age									−0.01 (0.00)	−0.02; 0.00	0.107	0.01
Household size									−0.14 (0.05)	−0.24; −0.05	0.003**	<0.01
Education [university degree]									0.12 (0.11)	−0.10; 0.35	0.277	0.01
Random effects												
Level 1 intercept σ^2^	0.21		0.20		0.21		0.22		0.14			
Level 2 intercept σ^2^	0.13		0.12		0.13		0.12		0.12			
Goodness of fit												
-2LL	471.02		468.31		466.96		444.64		415.20			
∆χ2			2.71°		1.35		22.32***		29.44***			
∆*df*			1		3		3		4			

For all models *n* of level 1 (persons) = 126. *N* of level 2 (households) = 92. Intervention group 4 is the reference group. Time [0] represents the pre measure and is the reference group for time of measure, time [1] represents the post-measure. °*p* < 0.10. *p < 0.05. **p < 0.01. ***p < 0.001.

**Table 12:** Mixed-model regression of ingroup identification as interaction of time (t0/ t1/t2) and the intervention groups, including control variables.

	Ingroup identification											
Predictor	Model 0		Model 1		Model 2		Model 3		Model 4			
	*b (SE)*	*p*	*b (SE)*	*p*	*b (SE)*	*p*	*b (SE)*	*p*	*b (SE)*	95% CI	*p*	f^2^
Fixed effects												
Intercept	2.65 (0.06)	<0.001***	2.62 (0.07)	<0.001***	2.59 (0.12)	<0.001***	2.50 (0.12)	<0.001***	2.12 (0.15)	1.82; 2.43	<0.001***	
Time [1]			0.09 (0.06)	0.122	0.09 (0.06)	0.127	0.11 (0.11)	0.331	0.10 (0.11)	−0.11; 0.32	0.349	0.17
Time [2]			0.04 (0.07)	0.565	0.05 (0.07)	0.524	0.27 (0.12)	0.029*	0.26 (0.12)	0.02; 0.50	0.033*	
Intervention group 1					0.01 (0.16)	0.943	0.11 (0.17)	0.517	0.05 (0.16)	−0.27; 0.36	0.773	0.18
Intervention group 2					0.08 (0.16)	0.625	0.03 (0.17)	0.847	−0.02 (0.16)	−0.34; 0.29	0.888	
Control group 3					0.04 (0.08)	0.635	0.25 (0.10)	0.010*	0.26 (0.10)	0.07; 0.45	0.007**	
Time[1]: intervention group 1							0.11 (0.17)	0.520	0.09 (0.16)	−0.23; 0.42	0.581	0.18
Time[2]: intervention group 1							−0.51 (0.18)	0.005**	−0.52 (0.18)	−0.87; 0.17	0.004**	
Time[1]: intervention group 2							0.36 (0.16)	0.029*	0.33 (0.16)	0.02; 0.65	0.039*	
Time[2]: intervention group 2							0.01 (0.17)	0.948	−0.01 (0.17)	−0.34; 0.33	0.949	
Time[1]: control group 3							−0.36 (0.15)	0.017*	−0.37 (0.15)	−0.66; −0.08	0.014*	
Gender [female]									0.47 (0.11)	0.25; 0.68	<0.001***	<0.01
Age									−0.01 (0.00)	−0.01; 0.00	0.137	0.01
Household size									−0.14 (0.05)	−0.23; −0.04	0.004**	<0.01
Education [university degree]									0.12 (0.11)	−0.10; 0.35	0.286	0.01
Random effects												
Level 1 intercept σ^2^	0.22		0.21		0.21		0.23		0.14			
Level 2 intercept σ^2^	0.13		0.13		0.13		0.12		0.12			
Goodness of fit												
-2LL	553.12		550.73		550.28		517.24		487.84			
∆χ2			2.39		0.44		33.05***		29.39***			
∆*df*			2		3		5		4			

For all models *n* of level 1 (persons) = 126. *N* of level 2 (households) = 92. Intervention group 4 is the reference group. Time [0] represents the pre measure and is the reference group for time of measure, time [1] represents the post-measure, and time [2] the follow-up. °*p* < 0.10. *p < 0.05. **p < 0.01. ***p < 0.001.

#### 4.2.3 Exploratory analysis

SIMPEA implies, that motivation (here represented by sufficiency attitude), appraisal of a crisis and environmental goals influence pro-environmental behavior. These aspects were analyzed exploratively. Results are also displayed in Tables 7, 8. The variables sufficiency attitude (*b*  = 0.36 [0.14, 0.57], *p* < 0.001, *f^2^*  < 0.01), appraisal of the packaging waste crisis (*b*  = 0.57 [0.14, 1.02], *p* = 0.04, *f^2^* = 0.04) and the goal to do precycling (*b*  = 0.18 [0.05, 0.32], *p* = 0.004, *f^2^* < 0.01) directly predict precycling behavior. Further, appraisal (*b*  = 0.33 [0.12, 0.54], *p* = 0.002, *f^2^* < 0.01) and goal (*b*  = 0.08 [0.02, 0.14], *p* = 0.010, *f^2^*  < 0.01) predict reuse behavior. In terms of effect size, only the effect of appraisal on precycling seems relevant.

## 5 Discussion

The first goal of the HomeLab intervention was to promote precycling behavior in Berlin households by applying social identity-based strategies proposed to encourage pro-environmental behavior in a newly formed community. In addition, the intervention should create a community with which the participants can identify. Furthermore, the objective was to test whether the proposed social identity processes ingroup identification, precycling-friendly ingroup norms and collective efficacy beliefs predict precycling behavior.

### 5.1 Intervention effects on precycling behavior, reuse behavior and ingroup identification

The precycling behavior slightly increased in all groups. Surprisingly, this change was independent of the intervention condition. There are two explanations for this. First, it is possible, that before the intervention, all participants were prepared by the recruitment flyer to work on their precycling behavior during the study, and therefore the intervention had no additional effect on their precycling behavior, which may explain the insignificant interaction effect between time and group. The flyer for the study said “Take the experiment and become a precycling household! Berliners become active to avoid packaging.” With this in mind, even participants in the waiting control condition might have tried to improve their precycling during the waiting period, even though they were not explicitly asked to do so. Second, it is important to note that all households in all conditions had documented their packaging waste in the packaging diary before and after the intervention. This diary method of data collection may have provided an initial stimulus to reflect on habits that helped the participants to cross psychological boundaries of time and space (Van Lange et al., 2018) in relation to packaging waste. In other words, this method may have increased awareness and perceived responsibility for the packaging produced, thereby reducing the distance between isolated behaviors and the environmental impact of those behaviors (Van Lange et al., 2018; Heidbreder et al., 2022). As a result of this diary method, participants in all groups – including the waiting control condition – may have started to reduce their packaging waste. In that vein, data collection through the packaging diaries would already comprise an intervention (Caspers et al., 2023). While it is not possible to determine exactly why the participants’ precycling behavior increased, the analysis by Caspers et al. (2023) shows that over the course of this intervention, also the actual quantity of packaging waste in the households as well as the associated carbon footprint decreased in all groups. Importantly, this implies that self-reported precycling is related to actual waste generation and environmental impact in this study.

While the different types of intervention did not have a different impact on the precycling behavior in the conditions, the intervention program in group two was successful in increasing the participants’ reuse behavior, both in the short and the long term. This difference may be explained as follows. In terms of content the participants in group two received specific self-reflection impulses and material interventions [Precycling Starter Kit with reusable and returnable meal boxes (see Section 3.1.2)] to stimulate reuse behavior. Furthermore, they attended interactive group discussions to exchange on their reuse experiences. The increased reuse behavior in group two cannot be clearly attributed to a specific intervention. Given the finding that also ingroup identification increased significantly in group two, it is possible that both the behavioral intervention and the social processes positively influenced reuse behavior. Future intervention studies could examine these intervention approaches in separate groups to investigate the processes underlying the observed effects.

With regard to the measurement of these intervention effects, it is also worth to look at the scales. The precycling behavior was measured using the validated behavioral scale by Klug and Niemand (2021), which does not consider reuse behavior explicitly. However, since the importance of reuse as subset of precycling had been highlighted before (Wenzel and Süßbauer, 2021), reuse behavior was implemented as an intervention element in group two and assessed with specific items. These reuse items appear to capture the effects of the behavioral intervention on reuse more specifically than the more general precycling scale. Therefore, in this case, it was important to capture the behavior as concretely as possible by also using the reuse items in order to map the effects of the intervention.

Through the intervention, there was a change in identification with the new precycling community that was built. While participants in group two identified more strongly with the community, the participants in the control group identified less strongly after the intervention period. This seems reasonable given the respective group formats. In group two, the social interaction between the participants was most extensive (group discussions). In contrast, the participants of the control group had no interaction at all during this period. Previous studies already indicated that interaction in small group discussions could activate social identity and promote collective identities (Postmes et al., 2005; Kuppens et al., 2013; Thomas et al., 2016a). In view of the items used to measure ingroup identification in this study (e.g., “Members of the Precycling-HomeLabs have a lot in common with each other”), one can easily envision that the group discussion about the participants’ experiences contributed to the identification with each other. In line with this, participants of group two stated to have become aware of commonalities with the other group members and shared goals due to the discussions. A female participant, who lives in a single household, said “It was nice to see that other people also have similar problems. So also in this group discussion, I thought that was great because we all had the same problems and we all talked about it like that.” Another female participant, who lives in a couple household, commented “I found it valuable to simply deal with something new and that it was a group where everyone had the same goal.” In light of these results, interaction and discussions on group-relevant topics might provide inspiring places of social identification and community building.

### 5.2 The role of social identity processes in precycling behavior

The social identity processes of ingroup identification, ingroup norms, and collective efficacy beliefs were expected to predict precycling and reuse behavior in the context of this intervention study. However, contrary to this expectation, only collective efficacy beliefs toward the HomeLab community predicted precycling behavior, but not reuse behavior.

There are several possible reasons why higher identification with the precycling community does not result in higher precycling and reuse activity. Previous studies that inspired this idea had only found that identification influenced behavioral intentions to buy organic food (Masson and Fritsche, 2014) and recycling (Nigbur et al., 2010) and had not looked at reported behavior, as in this study. Possibly, ingroup identification with a pro-environmental group influences behavioral intention but does not always result in pro-environmental behavior. Furthermore, the applied scale by Roth and Mazziotta (2015) has previously been tested for identities related to membership in large social categories (e.g., gender, nationality). According to them, it remains to be seen whether it is also suitable for small groups. The HomeLab group would probably be classified as small to medium. Perhaps the scale was less appropriate for this type of group, which contributed to the unexpected finding that identification did not predict precycling behavior. Further, Masson and Fritsche (2014) suggested distinguishing the effects of different aspects of ingroup identity, namely group-level self-investment and self-definition. Likewise, Roth and Mazziotta (2015) noted that the role of the specific components of social identification in explaining intragroup phenomena is not well studied yet. To assess whether self-investment and self-definition would affect the prediction of precycling behavior and reuse behavior differently, I tested the influence of the components on precycling behavior and reuse behavior separately. However, the results did not differ substantially from the model which considers the full social identification scale by Roth and Mazziotta (2015).

The results regarding social norms are also unexpected. Whereas previous studies revealed social norms to positively impact plastic-related and waste-minimization behavior (de Groot et al., 2013; van der Linden, 2015; Heidbreder et al., 2019, 2022; Kaplan Mintz et al., 2019), the HomeLab data do not mirror these finding. One explanation for this finding is that personal norms may be more important than social norms in the context of precycling, as in Heidbreder et al. (2022), because precycling is still a niche activity. The HomeLab participants were already motivated to practice precycling behavior. Because their personal belief was strong, they could not be dissuaded even if others around them did not practice precycling. In contrast, people who are not committed to precycling yet may be more responsive to the influence of social norms. Furthermore, compared to other social influence approaches (e.g., face-to-face interaction, social networks), the use of social norms is less effective (Abrahamse and Steg, 2013). Perhaps the effects of social interaction and community setting were more important in this study. As hypothesized, collective efficacy beliefs predict precycling behavior. That is, participants who believed in the HomeLab group’s ability to promote precycling and help solve the packaging waste crisis reported more precycling behavior than participants with lower collective efficacy beliefs.

The significance of sufficiency attitude as a motivator of pro-environmental behavior demonstrated here contributes new insights to psychological theorizing. The role of sufficiency attitude for pro-environmental action compared to other motivating factors could be the subject of future research. Regarding goals, it should be mentioned that participants were communicated the collective goal to do precycling during the HomeLabs, but the questionnaire assessed the individual goal (“I plan to avoid food packaging waste”). The individual goal was measured instead of the collective target, because not all participants shared their precycling goals with each other and therefore could not evaluate whether the other participants share the collective goal. Future studies could deepen the understanding of the psychological processes underlying the observed effects. For example, it would be interesting to examine the interplay of social identity and social interaction and group dynamics among the household members as subgroups, as noted by Thomas et al. (2016b).

### 5.3 Limitations and future directions

#### 5.3.1 Sample and method

First, with regard to the sample, it is worth mentioning that it was comparatively large for an intervention study of this complexity and that it represents heterogenic life constellations and sociodemographic characteristics. Nevertheless, a larger sample size for the analysis would have been desirable in terms of power. As elaborated by Snijders (2005) in multilevel models, power can be considered as a consequence of the standard error of estimation. In this sense, the fact that the standard error in the here presented models remains small as variables are added, is an indication that the models could withstand the complexity of the analyses. Nonetheless, it should be noted that some existing effects may not have been detected. Furthermore, the sample composition is not representative of the German population but has a bias towards female and academic participants. Participation in the study was voluntary and most participants were motivated to work on the topic. Hence, the results are not generalizable to populations with less internal motivation. At baseline, household types were not perfectly balanced across the different intervention groups although the assignment of the participants to the respective groups was randomized with respect to equal composition relation (stratified randomization). This is due to the dropout/non-participation of some participants and changes in household composition between the initial application for the study and the beginning of data collection. Furthermore, persons who applied for the study animated household members to participate, this influenced the composition of the overall sample additionally. Important to note is that all participants were living in Berlin. It can be assumed that everyday life in a metropolis is different from that in a small town or the countryside, in terms of precycling opportunities and social interaction. However, the decision to focus on Berlin was made to ensure that all participants have the most similar infrastructure available for the use of reusable packaging systems and waste disposal. Similar studies in non-metropolitan regions and in other countries would be important to generalize the results.

Second, as in many other psychological investigations, the limitations of self-report measures are evident in our study. Social desirability bias, memory lapses or inattention are just some of the sources that affect data quality on the part of participants; and furthermore, standardized questionnaires offer only limited access to very individual contexts (Sudbury-Riley and Kohlbacher, 2016). It is also important to note that, over the course of the study, the research team had personal and digital contact with many of the participants via email to coordinate the different parts of the study (e.g., answering organizational questions, contact during the webinars, scheduling an interview). This contact might have had effects on the participants’ behavior and response tendencies during the study, e.g., through effects of social desirability. These effects might have been stronger than in survey studies without contact. On the other hand, it could also be that these contacts led to an even stronger identification of the participants with the Precycling-HomeLabs.

Third, some of the scales used need to be discussed. The scale by Klug and Niemand (2021) used in this study measures precycling as a lifestyle that aims to conserve resources by avoiding packaging waste and making sustainable consumption choices. But does not consider different behavioral strategies that contribute to waste minimization such as reuse behavior. Since one part of the intervention focused on reuse behavior, a second scale was used to specifically assess reuse behavior. Indeed, the reuse scale seems to capture the intervention effect in group two. Unfortunately, Cronbach’s alpha for this self-constructed reuse scale was quite low, possibly because the scale covers a range of different reuse behaviors but was evaluated as a one-dimensional scale in the analysis. Alpha was not improved by removing certain items, so based on conceptual considerations and appropriateness, I included all six items in the final analysis. Future studies might improve this measure. Nevertheless, it was important to integrate this reuse scale as a measure of behavior change, because the precycling scale by Klug and Niemand (2021) is more likely to measure precycling lifestyle but may be less sensitive to behavior change. For even more valid conclusions about behavior and its change, future research would benefit from supplementing questionnaire data with actual behavioral data, such as data on actual waste generated. Also, it should be noted that the internal consistency of the rating scale for appraisal was not high (*α*_t0_ = 0.63). This may be because the items deal with very different issues, some of which are less addressed in public discourses (e.g., “species extinction through packaging in the environment”) and therefore less likely to be on people’s minds. For the regression analysis, a low Cronbach’s alpha means that the measurements of these variables is less precise and there is more variability in the measurements due to measurement error. Because of this higher variability in the data, the estimators are less precise, significance is more difficult to achieve, and the power of the analysis is lower. Despite these limitations, reuse behavior and valuation were significant in some models. This shows that these effects were strong enough to be detected.

Fourth, the rationale for having a waiting control group was that this allowed the effect of the waiting period and the effect of the webinars to be separated. In the analysis, the waiting period and the delayed intervention (webinar) could be interpreted as two different conditions. Thereby, a fourth intervention group condition was created artificially representing the effect of the delayed intervention (webinars). This fourth group represented the reference group in the analysis because due to this design, the effect of the provided information in the webinars could be controlled in all groups. This control was appropriate because the contents of the webinars were delivered by external experts and the formats were interactive and dynamic, which means that the format could not be planned exactly in advance in terms of communication and teaching strategies.

Fifth, it is in the nature real-world interventions that the results are influenced by many factors that have not even been discussed in this section. This makes it difficult to draw clear causal conclusions about the exact processes underlying the observed effects, such as which method had the greatest impact on social identity and behavior, or the extent to which individuals within the households influenced each other. Nevertheless, this complex study design reflects the interacting dimensions of everyday life better than isolated laboratory experiments. However, one aim was to find out whether the intervention helps to promote precycling and to translate the findings into practical knowledge. This makes it all the more important to test measures that might have been proven effective under controlled conditions in the real world.

Sixth, the study was conducted during the COVID-19 pandemic. During the study period, from May to November 2021, policies were in place which could have had an impact on everyday life and food habits in general, and specifically on the domestic use of food packaging, for example, closed restaurants or mandatory testing. Because of the pandemic situation, the HomeLabs were carried out completely digitally. Some participants reported back that the digital exchange with the participants was sometimes difficult and that it was hard for them to develop a sense of community in this context *“I think I would have liked to get in contact more with the other participants of the HomeLabs. Of course that was only possible to a very limited extent through Corona.”* (male participant, living in a flat share). Perhaps, the digital formats limited the potential for ingroup identification. On the other hand, the digital format made participation in the study more accessible, so it may have been possible to reach more people.

#### 5.3.2 Practical implications and conclusion

In conclusion, some ideas on how to support precycling and how to translate the findings into everyday activities are outlined. Although these Precycling-HomeLabs are a complex intervention package that required a lot of time and human resources, this need not to be a limitation for further implementation. Some of the implemented methods appear to have been more effective than others in increasing domestic precycling and are relatively easy to implement with little or no additional support.

The HomeLab seems to have supported all participants in implementing precycling behavior and reducing packaging waste. Especially the packaging diaries seem to be a way to become aware of one’s own behavior and to initiate change (Van Lange et al., 2018; Caspers et al., 2023). Starting a precycling journey as a group or alone can benefit from this pre-structured self-observation via a waste-protocol. The diary sheets come with background information and can be customized to meet specific interests, such as starting with documenting and then reducing a particular type of packaging.

Also, addressing reuse behavior as a specific strategy for precycling has proven to be effective. The very specific tasks of where and how to try out reuse behavior seem to have supported this process. The workbook provided guidance on this and can be used without prior knowledge by lay people and practitioners.

Within four weeks, the HomeLab community became a point of identification for the participants in the second intervention group. This means, that a new top-down community can quickly become an inspiring place of identification and pro-environmental behavior, even in a digital environment. The small-group activities, such as the group discussion, probably played an important role in developing a sense of community and unity around the common theme of precycling. Such a community, sharing a pro-environmental goal and norms, could then help the members build a strong environmental identity and transfer it to other groups and actions (Fielding and Hornsey, 2016). A promising approach would be to address existing communities and groups in social networks (e.g., neighborhood networks) that are open to the precycling-topic, and to create spaces for the exchange of ideas and experiences, as well as for the promotion of joint activities.

Furthermore, the study affirms the relevance of a number of SIMPEA elements that should be strengthened to promote domestic precycling. First of all, it is important to know, that we are dealing with a global crisis because of the masses of packaging waste have negative consequences in many areas. This appraisal of the crisis is already high (The United Nations Environment Assembly, 2022) and should remain high by continuing to report on it. Then, to increase people’s collective belief in their ability to counteract this problem, public communication could emphasize the impact of domestic precycling in transforming food supply chains to conserve resources. The goal of reducing packaging waste also contributes to implementation and could be supported by special campaigns in which joint efforts are made to put the goal into action (e.g., plastic free juli, Heidbreder et al., 2020). Last but not least, having a positive attitude towards a sufficiency-oriented lifestyle results in more precycling behavior. The acceptance and support of decisive reduction of resource usage should be promoted by emphasizing the benefits of this approach. Addressing these elements will help to promote precycling action.

While the contribution to resource conservation made by household-focused interventions is important, their impact is limited with regard to the extent of the waste crisis. This crisis will not be solved by behavioral interventions alone but strongly demands holistic approaches and systemic transformation. Thus, sustainable use of resources will involve changes and solutions in terms of a transformed production and food supply system, a social and structural environment that favors precycling and, ultimately, less consumption (Wilts and von Gries, 2015; Wiefek et al., 2021; Sattlegger and Süßbauer, 2022).

## Data availability statement

The datasets analyzed for this study can be found in an online repository at https://osf.io/v7j6w/.

## Ethics statement

Ethical approval was not required for the studies involving humans because participation was voluntary, there were no risks to participants’ physical or psychological integrity, participants were informed and consented to privacy conditions, there was no deception, and data were analyzed anonymously. The studies were conducted in accordance with the local legislation and institutional requirements. The participants provided their written informed consent to participate in this study.

## Author contributions

KW: Writing – review & editing, Writing – original draft, Methodology, Investigation, Formal analysis, Data curation, Conceptualization.

## Footnotes

^1^ https://aspredicted.org/blind.php?x=3d5pm8
