# Supplementary material for: Promoting collective precycling behavior: results from a group intervention with Berlin households in Germany
Source: Front Psychol. 2024 Jun 11;15:1340305. doi: 10.3389/fpsyg.2024.1340305 (PMC11197976; doi:10.3389/fpsyg.2024.1340305)
Supplement: Supplementary file 1 [file Data_Sheet_1.docx]

**Appendix A: Summary of the Content of the Webinars**

| *Summary of the Content of the Webinars.* | |
| --- | --- |
| Topic 1:  Precycling in everyday life | Webinar one was an introduction to precycling in everyday life with a focus on prevention and reuse, with a presentation of the waste hierarchy, of waste in the kitchen and at the grocery store, the experts provided specific options and tips for precycling in different daily contexts as well as recommending for reuse-projects in Berlin. |
| Topic 2:  Precycling by using tap water | Webinar two dealt with the precycling potential of tap water, first, the experts created problem awareness (increasing resource consumption through water bottles), then highlighted the good quality of tap water in Germany, explained five positive arguments for drinking tap water and motivated the participants to drink tap water throughout the webinar. |
| Topic 3:  Shopping in zero-waste stores | In webinar three, two owners of a zero waste shop talked about their personal motivation for their project, presented their concept, took the participants on a digital store tour, talked about the potentials of this concept, explained how zero waste shopping will work and gave tips on useful behavioral strategies. |
| Topic 4:  Recycling: When packaging was unavoidable | Webinar four addressed the question of how resources from food packaging can be reused in the best possible way through correct recycling when precycling has not been possible. |
