# Supplementary material for: Promoting collective precycling behavior: results from a group intervention with Berlin households in Germany
Source: Front Psychol. 2024 Jun 11;15:1340305. doi: 10.3389/fpsyg.2024.1340305 (PMC11197976; doi:10.3389/fpsyg.2024.1340305)
Supplement: Supplementary file 2 [file Data_Sheet_2.docx]

**Appendix B:** **Template of the Packaging Diary**

| Packaging Diary protocol sheet | | | | | Week | | | | Household Code | |
| --- | --- | --- | --- | --- | --- | --- | --- | --- | --- | --- |
| **Completion note:** Please document your packaging waste every day. Please use one line per packaging and go through the steps in the instructions for each and enter the information in the sheet. If the product packaging consists of several parts, please enter them together on one line. | | | | | | | | | | |
| No. | Step 1: Day of the week | Step 2: Product description | | Step 3: Packaging material type | | | Step 4: Material and recycling code | | Step 5: Disposal route and further use of the packaging | |
|  |  | What food has the packaging packed? | Weight or volume of the packed product  [x kg or x L] | What type of packing material is this? [Packing material type] | Comment | Quantity | What material is the packaging made of? [Material group] | Is there a material note or recycling code on the packaging? If yes, which one? [Recycling code/material notice] | What did you do with the packaging after use? [Disposal route, A1-5; B1-2;C] | Did you reuse or continue to use the packaging? If yes, for what? [Area of use] |
| 1 |  |  |  |  |  |  |  |  |  |  |
| 2 |  |  |  |  |  |  |  |  |  |  |
| 3 |  |  |  |  |  |  |  |  |  |  |
| 4 |  |  |  |  |  |  |  |  |  |  |
| 5 |  |  |  |  |  |  |  |  |  |  |
| 6 |  |  |  |  |  |  |  |  |  |  |
| 7 |  |  |  |  |  |  |  |  |  |  |
| 8 |  |  |  |  |  |  |  |  |  |  |
| 9 |  |  |  |  |  |  |  |  |  |  |
| 10 |  |  |  |  |  |  |  |  |  |  |
| 11 |  |  |  |  |  |  |  |  |  |  |
| 12 |  |  |  |  |  |  |  |  |  |  |
| 13 |  |  |  |  |  |  |  |  |  |  |
| 14 |  |  |  |  |  |  |  |  |  |  |
| Step 6. Notes on special events during the week | |  | | | | | | | | |
|  |  |  |  |  |  |  |  |  |  |  |
|  |  |  |  |  |  |  |  |  |  |  |
|  |  |  |  |  |  |  |  |  |  |  |
|  |  |  |  |  |  |  |  |  |  |  |
|  |  |  |  |  |  |  |  |  |  |  |
