# Supplementary material for: Promoting collective precycling behavior: results from a group intervention with Berlin households in Germany
Source: Front Psychol. 2024 Jun 11;15:1340305. doi: 10.3389/fpsyg.2024.1340305 (PMC11197976; doi:10.3389/fpsyg.2024.1340305)
Supplement: Supplementary file 3 [file Data_Sheet_3.docx]

**Appendix C: Guideline Group Discussion**

**Introduction**

First of all, we would like to start with a brief introduction round. For this, we would like to know:

- Your name
- Who do you live with?
- What is your biggest packaging sin?

Afterwards, please pass the conversation to the person on the top left of your screen. My colleague and I will begin and then pass it on to you.

**Experiences with Workbooks**

Firstly, we would like to talk about your experiences with the workbooks.

- Starting with the picture you brought along. Please share it via screen sharing and briefly explain why you chose it.
- Please also share your general experiences with the workbooks:
- How did you experience filling in the workbooks? Were you able to find time to complete it?

**Packaging Volume and Avoidance**

During the first week of HomeLabs, you focused on your packaging volume and potential avoidance strategies. Please tell the others about your results.

- In which area did you produce the most packaging waste?
- Where is it still particularly difficult? What is your biggest challenge (personally or
  as a household)
- What have you planned personally or as a household where you want to avoid packaging?
- How do you want to proceed with this?
- Do you think that you have been able to reduce packaging in this area? How realistic is your plan?
- If already implemented: Who or what helped you to do this?
- Do you have any tips for avoiding packaging waste or questions that you would like to share with us?
- Who has made similar experiences?
- How did the others solve this?
- Do you have any questions for the other participants?
- Do you think you can avoid packaging waste in the future/after HomeLabs?
- What could be obstacles?
- Would it be possible time-wise?
- Would it work in terms of coordination with other household members?

**Reuse-Interventions**

Now we come to the reuse tasks. In the last few weeks, you have tried out three different
reusable containers [tap water/ food for consumption on the go/ unpacked shopping] and
solved the corresponding tasks.

- How have the reusable experiments affected your everyday life?
- Related to coordination with other household members?
- Related to coordination with other activities, e.g. work, childcare?
- Which task or with which reusable container did it work well - which rather not? Could you please tell us a little about your experiences?
- How satisfied are you with your experiments? Did anything obstruct them?
- What do you think could be the reason for that?
- What about the other reusable container options?
- How did it go with the others? Did you have similar experiences or completely different ones?
- If it worked well: What helped you?
- What would you recommend to other households to make the use of reusable systems work?
- What would need to happen for you to better integrate reusable systems into your everyday life?
- Regarding your household organization?
- In terms of infrastructure/supply?
- Socially/politically?

**Conclusion**

The group discussion is coming to an end. Thank you for actively participating in the discussion.

- Do you have any additions to what has been said so far? Are there any points that are important to you but have not been mentioned yet?
- If you had to summarize your most important insight from the HomeLabs in one sentence, what would it be?
- Do you have any further questions about the upcoming process of the HomeLabs or any wishes for us?

Thank you for your participation!
