# Supplementary material for: Promoting collective precycling behavior: results from a group intervention with Berlin households in Germany
Source: Front Psychol. 2024 Jun 11;15:1340305. doi: 10.3389/fpsyg.2024.1340305 (PMC11197976; doi:10.3389/fpsyg.2024.1340305)
Supplement: Supplementary file 4 [file Data_Sheet_4.docx]

**Appendix D: Constructs Used in the Questionnaire**

| Construct | Measure | Time of measurement |
| --- | --- | --- |
| Ingroup Identification | We are all members of different groups and categories. For the following statements, please indicate how you subjectively feel about being a member of Precycling-HomeLabs. We use "Precycling-HomeLabs" here to refer to all participants* in the Precycling-HomeLabs study. There are no right or wrong answers to this question; we are only interested in your personal, honest assessment. To what extent do you agree with the following statements?  ingroupidentification_01_cen: I often think about the fact that I am a member of the Precycling-HomeLabs.  ingroupidentification_02_cen: The fact that I am a member of the Precycling-HomeLabs is an important part of my identity.  ingroupidentification_03_cen: Being a member of the Precycling-HomeLabs is an important part of how I see myself.  ingroupidentification_04_sol: I feel a bond with members of the Precycling-HomeLabs.  ingroupidentification_05_sol: I feel solidarity with members of the Precycling-HomeLabs.  ingroupidentification_06_sol: I feel committed to the members of the Precycling-HomeLabs.  ingroupidentification_07_sat: I am glad to be a member of the Precycling-HomeLabs.  ingroupidentification_08_sat: It is pleasant to be a member of the Precycling-HomeLabs.  ingroupidentification_09_sat: Being a member of the Precycling-HomeLabs gives me a good feeling.  ingroupidentification_10_ste: I have a lot in common with the average member of the Precycling-HomeLabs.  ingroupidentification_11_ste: I am similar to the average member of the Precycling-HomeLabs.  ingroupidentification_12_ste: I am a typical member of the Precycling-HomeLabs.  ingroupidentification_13_hom: Members of the Precycling-HomeLabs have a lot in common with each other.  ingroupidentification_14_hom: Members of the Precycling-HomeLabs are very similar to each other.  ingroupidentification_15_hom: Members of the Precycling HomeLabs share many characteristics in common.  (1 = ‘strongly disagree’ to 5 = ‘strongly agree’) | t0, t1, t1b, t2 |
| Precycling behavior | How much do you agree with the following statements?  precyclingbehavior1: I consciously buy unpacked food.  precyclingbehavior2: I buy sustainably produced food.  precyclingbehavior3: I try to avoid food packaging waste at all.  precyclingbehavior4: Compared to others I cause little food packaging waste.  precyclingbehavior5: I strictly reject superfluous food packaging waste.  (1 = ’completely disagree’ to 7 = ’completely agree’) | t0, t1, t1b, t2 |
| Reuse behavior | Please indicate how often you engage in these behaviors.  reuse1: I use my grocery bags multiple times.  reuse2: I reuse food packaging for the same purpose (e.g. paper bags for bread).  reuse3: I reuse food packaging for other purposes (e.g. tetra pack for crafting).  reuse4: I use reusable eating utensils when I am on the road (e.g. travel coffee mug, water bottle, reusable containers).  reuse5: To bring my shopping home, I use my own bag, not a store-provided one.  reuse6: I use my own containers to buy unpackaged food products.  (1 = ‘never’ to 5 = ‘very often’) | t0, t1, t1b, t2 |
| Goal | ‘To what extent do you agree with the following statement?’  Survey T0: goal_precycling: I plan to avoid food packaging waste  Survey T1: goal_precycling: During the last five weeks I have been trying to avoid food packaging waste.  Survey T2: goal_precycling: During the last three month I have been trying to avoid food packaging waste  (1 = ‘strongly disagree’ to 5 = ‘strongly agree’) | t0, t1, t1b, t2 |
| Descriptive social norms | DesNorms: ‘Please estimate the frequency with which the HomeLab participants try to minimize packaging waste when shopping food’  (1 = ‘never’ to 5 = ‘very often’) | t0, t1, t1b, t2 |
| Injunctive social norms | Please give your rating for the following statements.  InjNorms01: ‘If I reduced plastic while shopping, the HomeLab participants would …’ (1 = ‘approve’ to 7 = ‘disapprove’)  InjNorms02: ‘The HomeLab participants consider minimization of packaging waste while shopping food as (1 = ‘irrelevant’ to 7 = ‘very relevant’)’ | t0, t1, t1b, t2 |
| Collective efficacy beliefs | ‘This is about the collective impact of all participants during the Precycling-HomeLabs and of you as a participant in the Precycling-HomeLabs. How much do you agree with the following statements?’  collefficacybeliefs0:1 I believe that we, as members of the HomeLabs, can drive precycling.  collefficacybeliefs02: I believe that we, as members of the HomeLabs, can contribute to solve the environmental crisis through joint actions.  (1 = ‘totally disagree’ to 7 = ‘totally agree’) | t0, t1, t1b, t2 |
| Appraisal of the crisis | ‘Please rate the following aspects. Do you consider the following aspects as no problem, rather no problem, rather big problem, or very big problem?’  appraisal_aesthetic: aesthetic burden through packaging waste in the environment  appraisal_health: health impairment caused by packaging  appraisal_energy: energy and resource use of packaging production  appraisal_ocean: packaging waste in the ocean  appraisal_soil: packaging waste in soil  appraisal_extinction: species extinction through packaging in the environment  appraisal_cliamte: increase of climate change from packaging waste  (1 = ‘no problem’ to 5 = ‘very big problem’) | t0 |
| Sufficiency attitude | ‘Below you will read a series of statements. How much do you agree with the following statements?  sufficiency01: I find it desirable to possess few things only.’  sufficiency02 My comfort is more important than a frugal way of life.  sufficiency03: I think it is unnecessary to have this affluence of different products in our supermarkets.  sufficiency04: Through my lifestyle I want to use as few resources as possible.  sufficiency05: All the new things that are sold all the time are a big waste of resources to me  sufficiency06: I find it appealing to grow or produce as much food by myself as possible.  (1 = ‘strongly disagree’, 6 = ‘strongly agree’) | t0 |
| *Note.* t0 = pre, t1 = post (IG1 and IG2), t1b = post (WCG), t2 = follow-up. | | |
